# Impact of a *Plasmodium falciparum* AMA1 Vaccine on Antibody Responses in Adult Malians

**DOI:** 10.1371/journal.pone.0001045

**Published:** 2007-10-17

**Authors:** Alassane Dicko, David J. Diemert, Issaka Sagara, Moussa Sogoba, Mohamed B. Niambele, Mahamadoun H. Assadou, Ousmane Guindo, Beh Kamate, Mounirou Baby, Mady Sissoko, Elissa M. Malkin, Michael P. Fay, Mahamadou A. Thera, Kazutoyo Miura, Amagana Dolo, Dapa A. Diallo, Gregory E. Mullen, Carole A. Long, Allan Saul, Ogobara Doumbo, Louis H. Miller

**Affiliations:** 1 Malaria Research and Training Center, Department of Hematology, University of Bamako, Bamako, Mali; 2 Malaria Research and Training Center, Department of Parasitology, University of Bamako, Bamako, Mali; 3 Malaria Vaccine Development Branch, National Institute of Allergy and Infectious Diseases, National Institutes of Health, Bethesda, Maryland, United States of America; 4 Biostatistics Research Branch, National Institute of Allergy and Infectious Diseases, National Institutes of Health, Bethesda, Maryland, United States of America; London School of Hygiene & Tropical Medicine, United Kingdom

## Abstract

**Background:**

Apical Membrane Antigen 1 (AMA1) of *Plasmodium falciparum* merozoites is a leading blood-stage malaria vaccine candidate. Protection of *Aotus* monkeys after vaccination with AMA1 correlates with antibody responses.

**Study Design/Results:**

A randomized, controlled, double-blind phase 1 clinical trial was conducted in 54 healthy Malian adults living in an area of intense seasonal malaria transmission to assess the safety and immunogenicity of the AMA1-C1 malaria vaccine. AMA1-C1 contains an equal mixture of yeast-expressed recombinant proteins based on sequences from the FVO and 3D7 clones of *P. falciparum*, adsorbed on Alhydrogel. The control vaccine was the hepatitis B vaccine (Recombivax). Participants were enrolled into 1 of 3 dose cohorts (n = 18 per cohort) and randomized 2∶1 to receive either AMA1-C1 or Recombivax. Participants in the first, second, and third cohorts randomized to receive AMA1-C1 were vaccinated with 5, 20 and 80 µg of AMA1-C1, respectively. Vaccinations were administered on days 0, 28, and 360, and participants were followed until 6 months after the final vaccination. AMA1-C1 was well tolerated; no vaccine-related severe or serious adverse events were observed. AMA1 antibody responses to the 80 µg dose increased rapidly from baseline levels by days 14 and 28 after the first vaccination and continued to increase after the second vaccination. After a peak 14 days following the second vaccination, antibody levels decreased to baseline levels one year later at the time of the third vaccination that induced little or no increase in antibody levels.

**Conclusions:**

Although the AMA1-C1 vaccine candidate was well-tolerated and induced antibody responses to both vaccine and non-vaccine alleles, the antibody response after a third dose given at one year was lower than the response to the initial vaccinations. Additionally, post-vaccination increases in anti-AMA1 antibody levels were not associated with significant changes in in vitro growth inhibition of *P. falciparum.*

**Trial Registration:**

ClinicalTrials.gov NCT00343005

## Introduction

Due to the limited number of drugs available for treatment of *Plasmodium falciparum* malaria and increasing drug resistance, development of a malaria vaccine has become a global health priority. A vaccine is considered feasible given that individuals repeatedly exposed to the parasite gradually develop immunity to the clinical manifestations of infection. This resistance to clinical disease is partly mediated by antibodies to antigens expressed during the asexual blood stages of the *P. falciparum* life cycle [1], [2]. One such protein, the apical membrane antigen 1 (AMA1), has been shown to play a significant role in erythrocyte invasion [3]–[6].

Vaccination with recombinant AMA1 induces protection against homologous parasite challenge in both rodents and monkeys [7]–[11]. Due to the strain-specificity in antibody responses to AMA1 [9] and the sequence polymorphism of the AMA1 gene that exists among circulating strains of *P. falciparum* [12]–[14], the AMA1-C1 vaccine was designed as an equal mixture of the recombinant AMA1 proteins derived from the FVO and 3D7 clones of *P. falciparum,* in an attempt to induce protective immunity against diverse parasite strains present in endemic areas.

In a Phase 1 study of the AMA1-C1 vaccine adjuvanted with Alhydrogel conducted in malaria-naïve adults living in the United States, this vaccine was well-tolerated and elicited antigen-specific antibodies with biological activity against malaria parasites as judged by an in vitro growth inhibition assay (GIA) [15]. Greater antibody responses were seen after the third vaccination than after the first two vaccinations. No significant difference was observed in the antibody responses to the two highest dose concentrations tested (20 and 80 µg), although both were significantly higher than the lowest dose tested (5 µg), suggesting that a maximum response had been attained in this study population.

Due to these promising results, a clinical trial of the AMA1-C1 vaccine was performed in Mali, West Africa. The results of this study demonstrate that the AMA1-C1/Alhydrogel vaccine induces a significant humoral immune response in malaria-exposed individuals even after a single dose of vaccine that increases after a second vaccination given one month following the first. Surprisingly, administration of a third vaccination one year following the initial vaccinations did not result in increased antibody levels similar to those seen after the first two vaccinations.

## Methods

The protocol for this trial and supporting CONSORT checklist are available as supporting information; see Checklist S1 and Protocol S1.

### Study Site

The study was conducted in Donéguébougou, Mali, at a clinic operated by the Malaria Research and Training Center of the University of Bamako. Donéguébougou is a rural village of approximately 1300 inhabitants in which malaria transmission occurs mainly during the rainy season extending from June to November. Entomologic inoculation rates (EIR) as determined by the human landing catch method vary between zero in the dry season and 50 to 60 infective bites/person/month at the height of the rainy season in September and October [16].

The study was approved by the institutional review boards of the University of Bamako and the US National Institute of Allergy and Infectious Diseases, and was conducted under an investigational new drug application (BB-10944) to the US Food and Drug Administration.

### Participants

After obtaining permission from the village elders to conduct the study, 54 participants were enrolled into 1 of 3 cohorts. Written informed consent was obtained from healthy volunteers between the ages of 18 and 45 years. Volunteers were excluded if they had evidence of clinically significant systemic disease; were pregnant or breast feeding; had serological evidence of chronic hepatitis B or C infection; were receiving corticosteroids or immunosuppressive drugs; or had been immunized with a live vaccine within the previous month.

### Interventions

Recombinant AMA1-FVO and AMA1-3D7 were manufactured, mixed, and adsorbed to Alhydrogel (HCl Biosector) as described previously [9]. Each 0.5 mL dose contained either 5, 20, or 80 µg AMA1-C1 and 800 µg Alhydrogel. Potency studies in mice conducted every 6 months confirmed that all lots were stable and potent throughout the trial. Recombivax HB (Merck&Co.) was supplied in single-dose vials containing 10 µg of recombinant hepatitis B surface antigen adsorbed to amorphous aluminum hydroxyphosphate sulfate at a final volume of 1.0 mL. Vaccines were transported to the study site using temperature monitoring devices to ensure maintenance of the cold chain.

Within each cohort, participants were randomized to receive either AMA1-C1/Alhydrogel (n = 12) or Recombivax (n = 6), with the first, second, and third cohorts enrolled successively at three-week intervals. Participants randomized to AMA1-C1 received 5, 20 and 80 µg in the first, second, and third cohorts, respectively. Vaccinations were administered by intramuscular injection in the deltoid muscle on study days 0, 28, and 360.

The safety of study participants was monitored throughout the trial by an independent Malian physician. Interim safety reports were reviewed by a data and safety monitoring board prior to vaccination of the second and third cohorts, and before administration of the third vaccinations on study day 360.

### Objectives

The primary objective was to estimate the frequency of vaccine-related adverse events, graded by severity, for each dose of AMA1-C1 being tested. Secondary objectives included evaluation of the allele-specific antibody response to vaccination, assessment and comparison of the duration of antibody response to AMA1-FVO and AMA1-3D7, measurement of the inhibition of parasite growth as measured by the in vitro GIA to the FVO and 3D7 clones of *P. falciparum*, and determination of the relationship between anti-AMA1 antibody concentration, as judged by ELISA, and degree of in vitro growth inhibition of *P. falciparum* by GIA.

### Outcomes

Following vaccinations, volunteers were directly observed for 30 minutes and then evaluated 1, 2, 3, 7, and 14 days post-vaccination for evidence of local and systemic reactogenicity, and then monthly throughout the malaria transmission seasons following the second and third vaccinations until the end of the study approximately 6 months after the third vaccination. Injection sites were examined for erythema, swelling, and tenderness at the site of injection. Solicited systemic adverse events included fever or chills, headache, nausea, myalgia, and arthralgia. Adverse events were graded as either mild (easily tolerated), moderate (interfered with activities of daily living), or severe (prevented activities of daily living), and assigned causality relative to the study vaccine. Injection site erythema and swelling were graded as mild (>0 to ≤20 mm in diameter), moderate (>20 to ≤50 mm), or severe (>50 mm). Oral temperature was graded as mild (>37.5°C to≤38.0°C), moderate (>38.0°C to≤39.0°C), or severe (>39.0°C). A complete blood count, serum creatinine and alanine aminotransferase concentration were performed immediately prior to each vaccination as well on the third and fourteenth days following vaccination. Complete blood counts were also performed at the monthly visits during the malaria transmission seasons of each study year.

Anti-AMA1 antibodies were measured using a standardized ELISA [15]. ELISA plates were coated with AMA1-FVO, AMA1-3D7, or AMA1-L32, a *P. pastoris*-expressed recombinant protein based on the sequence of the L32 strain of *P. falciparum* (Genbank accession number EF221749) and that differs from the FVO and 3D7 antigens by 26 and 24 amino acids, respectively. Serial dilutions of a standard serum pool were included on each test plate to generate a standard curve, which was used to convert the absorbance of individual sera into antibody units. Participant samples from days 0 to 180 and from days 270 to 540 were tested at separate times.

Antigen-specific IgG subclasses were measured by a flow cytometric suspension array assay. Serum samples were mixed with microspheres coupled to AMA1 (Luminex Corporation). Mouse anti-human IgG subclass antibodies (anti-human IgG1, IgG2, IgG3, and IgG4) and a secondary donkey anti-mouse IgG phycoerythrin-labeled antibody (Jackson ImmunoResearch) were added to develop the reactions. Mean fluorescence intensities were detected by Luminex X-MAP using Bioplex software (BioRad).

IgG from vaccinated individuals were tested for their ability to inhibit *in vitro* growth of *P. falciparum* 3D7 and FVO parasites using a standardized GIA procedure [15], [17]. Values obtained with test samples were compared to those obtained from parasites incubated with a pool of malaria-naïve human serum and with uninfected red cells to obtain the percent inhibition in growth.

### Sample size

Although not powered to detect differences in the incidence of specific adverse events or immune responses between AMA1-C1 and Recombivax or between the different dose concentrations of AMA1-C1, a group size of 12 per dose concentration of AMA1-C1 was chosen to give a reasonable probability of detecting one or more serious or severe vaccine-related adverse events. A group size of 10 individuals would provide a power of 80% to detect an adverse event that occurs with a probability of 0.15; an extra 2 participants were recruited into each group in case of withdrawal or loss to follow-up.

In addition, each dose concentration of AMA1-C1 was compared to Recombivax, which served as a comparison for both immunologic and safety assessments. A total of 18 controls, 6 per each dose concentration of AMA1-C1, were enrolled to allow a 2∶1 ratio of AMA1-C1 to Recombivax within each cohort; thus, each of the 3 cohorts contained 12 volunteers receiving AMA1-C1 plus 6 receiving Recombivax for a total sample size of 54.

### Randomization

Within each cohort, participants were randomized to receive either AMA1-C1 or Recombivax by use of sealed envelopes labeled with a unique participant study number and containing the vaccine assignment. A set of 18 envelopes were prepared for each cohort such that 12 contained assignments to AMA1-C1 and 6 to Recombivax. Study numbers were assigned in the order that participants arrived at the clinic on the day of first immunization. Randomization envelopes were opened by a study pharmacist on the day of first immunization and immediately re-sealed after vaccine assignment.

### Blinding

Study participants and investigators who assessed outcomes were blinded to vaccine assignment. Access to randomization codes was limited to the study pharmacists. Syringe barrels were masked with opaque tape to disguise the contents, since the volumes of the two study vaccines were different. To reduce investigator bias, injections were administered by physicians who were not involved in post-vaccination safety assessments or study analysis.

### Statistical Methods

Differences in the proportion of individuals experiencing each adverse event (of any severity) between vaccine allocations (AMA1-C1 vs. Recombivax) and between vaccinations (first versus second, etc.) within each dose group were analyzed using Fisher's exact and McNemar's tests, respectively. The exact two-sided Cochrane-Armitage test for trend using rank scores was used to test for a dose effect for each solicited adverse event; for these analyses, the Recombivax group was assigned a dose of 0 µg of AMA1-C1.

Differences in the change in antibody level from baseline to post-vaccination time points were compared between AMA1-C1 and Recombivax using exact Wilcoxon rank sum tests within each cohort. Differences in change of antibody (days 0 and 42) between dose groups were analyzed using the two-sided Wilcoxon rank sum test, assuming the absence of an effect due to differing vaccination times for the cohorts in relation to the malaria transmission season (this assumption was not violated by a Kruskal-Wallis test for cohort effect on day 0 values [*p*>0.4]). Agreements between anti-AMA1-FVO, anti-AMA1-3D7, and anti-AMA1-L32 antibody responses were calculated using the random marginal agreement coefficient (RMAC) with the squared difference cost [18]. Correlations between percent growth inhibition and antibody responses were assessed by the Spearman rank test. The SAS (version 9.1; SAS), R (version 2.4.0; R Foundation for Statistical Computing) and STATA (version 8.0; StataCorp) software packages were used, and *p*<.05 was considered significant.

## Results

### Participant Flow and Baseline Data

**Figure 1:**
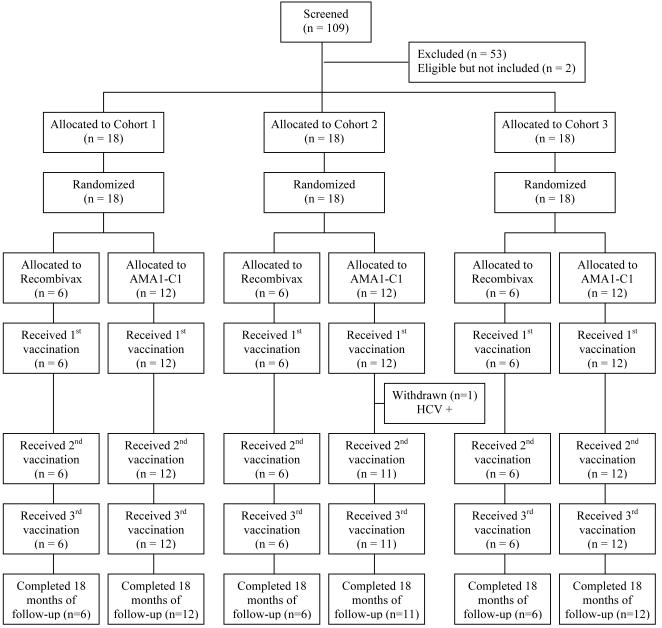
CONSORT Flow Chart.

One hundred and nine adults were screened for inclusion in the study, of whom 54 (45 males and 9 females) were enrolled (Figure 1). Reasons for exclusion were concurrent illness (n = 22), positive serology for chronic hepatitis B or C (n = 18), other abnormal screening laboratory tests (n = 9), intent to travel during the study period (n = 1), and history of significant allergy (n = 3); two volunteers were eligible but not enrolled.

The median age of participants was 30 years (range, 18 to 45). Vaccinations were initiated in May 2004; all cohorts received their second vaccination by the end of July 2004 before the onset of significant malaria transmission. The third vaccinations were administered in May and June 2005, immediately prior to the onset of that year's malaria transmission season. One subject, randomized to receive 20 µg of AMA1-C1/Alhydrogel was withdrawn after the first vaccination: this participant was anti-HCV antibody positive at screening but was inadvertently enrolled and received the first dose of vaccine before the error was discovered. Data from this subject were included in the safety but not the immunogenicity analyses. All other participants received all planned vaccinations and completed all scheduled study visits, and were included in all analyses.

### Safety

All vaccinations were well tolerated (Table 1). Mild pain and swelling were the most commonly observed injection site reactions, and there were no significant differences among the proportions of volunteers with these reactions between vaccines (AMA1-C1 vs. Recombivax) or between vaccinations within dose groups. Similarly, there was no dose response in the occurrence of injection site reactions except for injection site swelling after the first vaccination which occurred more frequently with increasing dose concentrations of AMA1-C1 (*p* = 0.03) . Solicited systemic reactions were uncommon, with mild to moderate headache being the most frequently observed event. There were no significant differences between AMA1-C1 and Recombivax or between successive vaccinations within dose groups, and no dose-response, in the incidence of systemic reactions.

**Table 1:** Solicited local injection site and systemic adverse events after vaccination with the AMA1-C1/Alhydrogel or Recombivax HB hepatitis B vaccines.

	Vaccination#1				Vaccination#2				Vaccination#3			
	AMA1-C1			Recombivax(n = 18)	AMA1-C1			Recombivax(n = 18)	AMA1-C1			Recombivax(n = 18)
	5 µg(n = 12)	20 µg(n = 12)	80 µg(n = 12)		5 µg(n = 12)	20 µg(n = 11)	80 µg(n = 12)		5 µg(n = 12)	20 µg(n = 11)	80 µg(n = 12)	
**Local**												
Pain	3	1	5	3	3	3	2	3	0	4	4	3
Swelling	1	4	6	3	5	4	5	9	3	1	1	5
Erythema	0	1	0	0	0	0	0	1	0	0	0	0
**Systemic**												
Fever	2	0	0	1	0	0	0	0	0	1	0	0
Headache	2	1	0	4	0	1	2	2	1	1	1	0
Nausea	0	0	0	1	0	0	0	0	0	0	0	1
Myalgia	0	1	0	0	0	0	0	0	0	0	0	0
Arthralgia	0	0	0	0	0	0	0	0	0	1	0	0

Data are number of study participants. All injection site reactions were mild whereas systemic reactions were either mild or moderate in intensity.

No serious adverse events, hypersensitivity reactions, or clinical laboratory abnormalities occurred that were related to vaccination with AMA1-C1/Alhydrogel.

### IgG responses to AMA1-3D7 and AMA1-FVO

**Figure 2:**
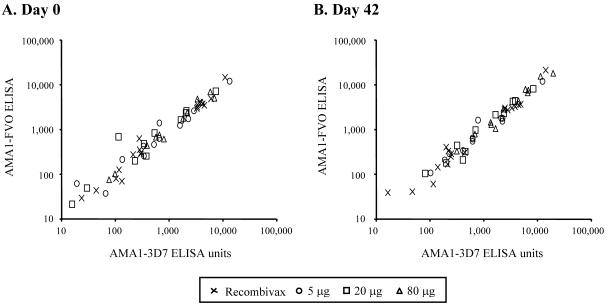
Comparison of pre-vaccination (A) and post-vaccination (B) antibody responses to AMA1-3D7 and AMA1-FVO. Sera collected on day 0 and 42 were assayed from all study participants. Concordance between the responses to the 3D7 and FVO alleles of AMA1 were highly significant on both days (RMAC, 0.97 on day 0 and 0.96 on day 42)

Prior to vaccination, antibodies to both AMA1-FVO and AMA1-3D7 were detectable in sera of individuals from all cohorts (Figure 2a). There was a highly significant concordance between the antibody responses to AMA1-FVO and AMA1-3D7 both before vaccination (RMAC, 0.97 [95% confidence interval {CI}, 0.95–0.98]; Figure 2a) and after vaccination (RMAC, 0.96 [95% CI, 0.94–0.98]; Figure 2b). Because of the high concordance between ELISA values for the two AMA1 antigens, data are only presented for AMA1-3D7. In all cases, similar data were observed for AMA1-FVO.

**Figure 3:**
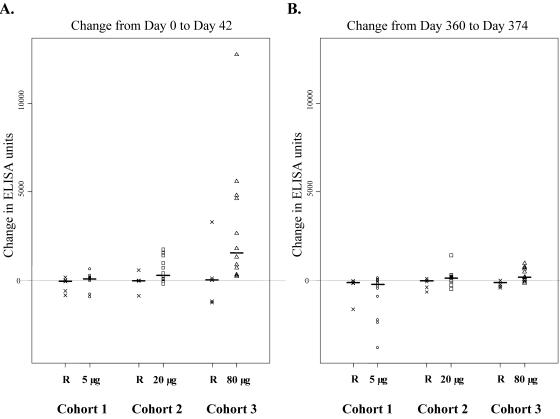
Change from baseline in anti-AMA1-3D7 antibody levels after the second and third vaccinations. Differences between study days 0 (day of vaccination 1) and 42 (14 days post-vaccination 2) (A) and between study days 360 (day of third vaccination) and 374 (14 days post-vaccination 3) (B) are shown. Bars represent the median change in antibody units against AMA1-3D7; R, Recombivax

There was a significant increase in antibody response from baseline to day 42 (14 days after the second vaccination) in both the 20 µg (median increase of 381 ELISA units; *p* = 0.01) and 80 µg (median increase of 1554 ELISA units; *p* = 0.007) groups compared to Recombivax within each cohort (Figure 3a), with the 80 µg group displaying a greater increase in antibodies to AMA1 than the 20 µg group (*p* = 0.03). However, 3 of 12 volunteers in the 80 µg group had a poor antibody response after the first two vaccinations (increases of 219 to 321 ELISA units; Table 2). The IgG subclass distribution for the AMA1 antibodies was unchanged by vaccination with AMA1-C1, and was comprised mostly of IgG1 and IgG3.

**Table 2:** Change in individual anti-AMA1-3D7 antibody levels after the second and third vaccinations with 80 µg of AMA1-C1/Alhydrogel.

Study Participant	Baseline Anti-AMA1-3D7 Antibody Level (ELISA Units)		Change in anti-AMA1-3D7 Antibody Level (ELISA Units)		
	Day 0	Day 360	Day 14^a^	Day 42^a^	Day 374^b^
38	78	157	277	1315	201
45	100	167	161	219	470
48	392	445	4	300	42
41	576	276	798	1793	655
44	670	592	584	698	135
49	815	442	59	868	22
50	1846	1477	1546	4795	716
40	2203	1976	1335	4619	169
37	3430	1366	758	2647	960
54	4182	2696	−605	321	−158
52	5901	3350	1233	5590	761
53	7037	7875	4739	12751	−102

a Compared to antibody level on day 0

b Compared to antibody level on day 360

**Figure 4:**
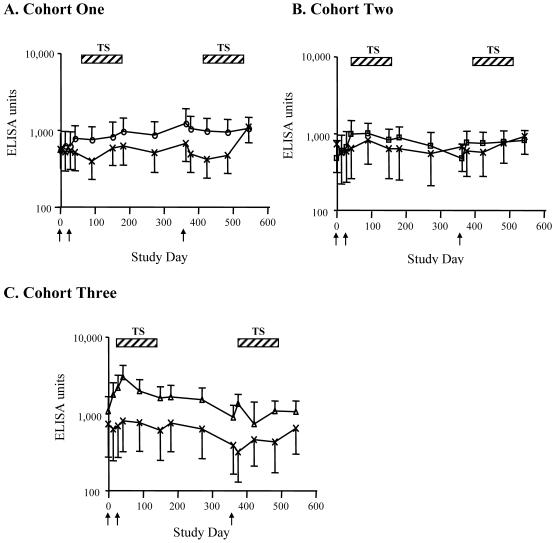
Longitudinal anti-AMA1 antibody responses in study participants from the first (A), second (B) and third (C) cohorts. Antibody units were measured by ELISA in sera collected on: day 0 (vaccination 1), day 14 (14 days post-vaccination 1), day 28 (vaccination 2), day 42 (14 days post-vaccination 2), day 90, day 180, day 270, day 360 (vaccination 3), day 374 (14 days post-vaccination 3), days 420, 480, and 540. Points represent the geometric mean antibody units against AMA1-3D7, error bars the standard error, and arrows the vaccination time points; TS, transmission season; °, 5 µg AMA1-C1; □, 20 µg AMA1-C1; Δ, 80 µg AMA1-C1; ×, Recombivax

**Figure 5:**
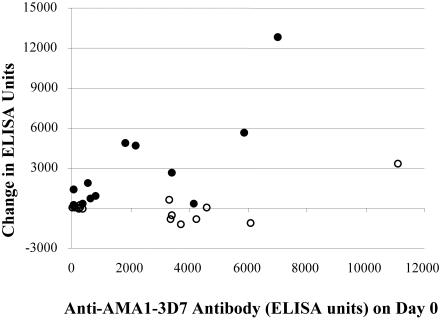
Post-vaccination changes in anti-AMA1-3D7 antibody levels compared to baseline levels. Change in anti-AMA1-3D7 antibody levels between study days 0 (day of vaccination 1) and 42 (14 days post-vaccination 2), are compared to the anti-AMA1-3D7 antibody level on day 0, in the Recombivax (open circles) and 80 µg AMA1-C1 (black circles) dose groups

The increase in AMA1-specific antibodies following the first vaccination with 80 µg of AMA1 was rapid and already apparent by day 14, consistent with boosting a memory response (Figure 4). To explore the effect of pre-existing antibody levels on the response to vaccination, the change in anti-AMA1 antibody levels was compared to pre-vaccination antibody levels (Figure 5). For the 80 µg AMA1-C1 group, there was a significant positive correlation between anti-AMA1 antibody levels on day 0 and the increase in antibody between days 0 and 42 (Spearman rank correlation, 0.66, *p* = 0.02; Figure 5).

After a peak in antibody levels two weeks following the second vaccination, anti-AMA1 antibody responses in the 80 µg AMA1-C1 dose group declined halfway to baseline by day 90 and returned to baseline by day 360, the day of the third vaccination (Figure 4). Participants were re-vaccinated approximately one year after the first two vaccinations and just prior to the start of the malaria transmission season. Following this third vaccination, a significant increase in anti-AMA1 antibody level was seen only in the 80 µg AMA1-C1 group when compared to Recombivax (*p* = 0.003), although the magnitude of this response was much lower than was seen after the first two vaccinations (Figure 3b): the median increase was only 185 ELISA units. Remarkably, the 7 participants with the largest increases in antibody after the first two vaccinations showed little or no response (i.e., less than 1000 ELISA units) to the third vaccination (Table 2). Furthermore, two volunteers (#40 and #53) who had high antibody levels after the second vaccination had no rise in antibody after the third vaccination (Table 2). Those who did not have appreciable responses after the first 2 vaccinations also did not respond to the third vaccination. There was no evidence of general immune suppression in the Malian volunteers because the third vaccination with Recombivax induced the expected rise in antibody levels to hepatitis B surface antigen (data not shown).

### IgG responses to AMA1-L32

To determine whether antibodies induced by vaccination would cross-react with other allelic variants of AMA1, antibodies to AMA1-L32 were assessed by ELISA. Two weeks after the second vaccination, antibody responses to AMA1-L32 increased significantly from baseline by a median of 533 ELISA units (*p* = 0.0004) in participants who received 80 µg AMA1-C1 compared to no increase in those receiving Recombivax. The increase in antibody to AMA1-L32 was lower than to AMA1-3D7 but the responses to the two alleles were still concordant (RMAC, 0.46 [95% CI, 0.34–0.56]).

### Growth inhibition assay (GIA)

Pre-vaccination IgG showed growth inhibition ranging from −4% to 67% for *P. falciparum* 3D7 (results for the 80 µg AMA1-C1 and Recombivax groups are given in Table 3). Overall, there was a statistically significant correlation between pre-vaccination anti-AMA1 antibody levels and percent growth inhibition of the 3D7 clone (Spearman rank correlation, 0.60; *p*<0.0001).

**Table 3:** *In vitro* growth inhibition of *Plasmodium falciparum* 3D7 by sera from recipients of Recombivax and 80 µg AMA1-C1/Alhydrogel.

Group	Study Participant	Anti-AMA1-3D7 Antibody^a^ (U)		GIA (% Inhibition)^b^		Change between Days 0 and 42	
		Day 0	Day 42	Day 0	Day 42	ELISA	GIA
*Recombivax*	2	181	178	21	19	−3	−2
	7	161	141	9	16	−20	7
	9	121	96	8	10	−25	2
	10	1460	1395	38	38	−65	0
	13	1137	943	54	50	−194	−4
	14	88	86	37	31	−3	−6
	19	129	114	36	34	−15	−2
	24	179	160	25	33	−19	8
	25	14	13	13	21	−1	8
	27	1143	1387	42	45	243	3
	28	1023	1337	40	47	313	7
	29	763	867	33	36	103	3
	39	78	61	14	11	−18	−3
	42	1003	931	42	39	−72	−2
	43	65	159	32	29	94	−4
	46	2944	2858	30	17	−87	−14
	47	32	36	12	2	4	−10
	51	3723	3371	55	61	−352	5
*AMA1-C1* (80 µg)	37	1607	2373	40	37	766	−3
	38	62	577	23	25	515	2
	40	750	2297	36	29	1547	−7
	41	156	439	20	18	283	−2
	44	352	733	36	35	382	−1
	45	53	103	42	33	50	−9
	48	237	302	17	5	65	−12
	49	423	662	25	29	238	4
	50	620	1745	67	62	1125	−4
	52	1635	4169	21	30	2534	9
	53	2579	5961	60	76	3382	16
	54	1349	2573	22	23	1224	1

a The ELISA unit values shown are the amounts of anti-AMA1 antibody in purified IgG added to test wells for the growth inhibition assay

b Percent inhibition of parasite growth compared to wells with equivalent concentrations of normal human serum

Following the second vaccination, no significant change in the growth inhibition of either *P. falciparum* 3D7 or FVO was achieved with any of the doses of AMA1-C1/Alhydrogel or Recombivax (Table 3). Additionally, among those receiving AMA1-C1, there was no statistically significant correlation between change (day 0 and 42) in percent growth inhibition and change in anti-AMA1 ELISA units in the IgG purified for the GIA (Spearman rank correlation, −0.06; *p* = 0.7). To investigate to what extent growth inhibition observed in individual sera was due to anti-AMA1 antibodies, day 42 samples from four individuals with high levels of anti-AMA1 antibody (one from each of the AMA1-C1 dose groups and one from the Recombivax group) were pre-incubated with AMA1 protein; minimal to no reversal of invasion inhibition was seen (data not shown).

## Discussion

### Interpretation

The results of this trial indicate that the AMA1-C1 blood-stage malaria vaccine adjuvanted with Alhydrogel is well-tolerated when administered to adult malaria-exposed volunteers living in Mali, does not result in significant vaccination-related adverse events, and induces significant anti-AMA1 antibody responses in this population. Significant responses were observed even after administration of a single 80 µg dose, suggesting that boosting of pre-existing immunity from prior exposure to natural infection is possible with this vaccine.

Given that the proposed mechanism of action of blood-stage malaria vaccines is through production of antibody against malarial antigens expressed on the surface of merozoites or infected erythrocytes, an important objective of this study was to test the ability of the AMA1-C1 vaccine to induce humoral immune responses in malaria-experienced individuals. Not surprisingly, pre-existing levels of anti-AMA1 antibodies were found in most study participants prior to vaccination, likely due to previous exposure to this antigen by natural infection. While vaccination with either Recombivax or 5 µg AMA1-C1 induced no significant increase in anti-AMA1 antibodies, a significant increase in responses to both AMA1-3D7 and AMA1-FVO was detectable after just one dose of 80 µg AMA1-C1 in the Malian volunteers.

However, the antibody response to vaccination with AMA1-C1 adjuvanted with Alhydrogel in semi-immune Malian adults differed from that observed in malaria-naïve adults in the US. In US volunteers, a recall response was induced upon vaccinating five months after the second vaccination although minimal levels of antibody were seen after the first two vaccinations regardless of dose [15]. In the Malian study, levels of AMA1-specific antibody increased after the first and second vaccinations in a dose dependent fashion, suggesting stimulation of antigen specific memory cells. However, only small increases in antibody were induced after a third vaccination of Malian adults administered one year later. Why did Malians who responded to the first two vaccinations, respond poorly to the third vaccination?

The first possibility is that the AMA1-C1 vaccine had lost potency either through degradation over time or because of inadequate transportation or storage conditions. However, this is unlikely, as biannual potency studies of the lots of vaccine used in this trial demonstrated continued ability to induce antigen-specific antibodies in animals. Damage during storage or transport is also unlikely because electronic temperature monitors recorded no temperature excursions. Furthermore, study participants who received the Recombivax vaccine, which was transported and stored in the same shipments, developed significant anti-hepatitis B surface antigen antibodies after the third vaccination.

The second possibility is that people living in malaria endemic areas do not generate long-lived B cell memory to AMA1. Arguing against this is the fact that some volunteers responded rapidly to the first vaccination with recall-like kinetics, indicating pre-existing B cell memory to AMA1 due to prior exposure to *P. falciparum*. Moreover, the vaccine itself was able to induce and recall memory B cells in vaccinated individuals living in the US [15].

What are the possible immunological mechanisms that might account for this effect? First, the Malian volunteers had naturally acquired circulating AMA1-specific antibodies prior to vaccination unlike the US volunteers who had none. It is therefore possible that immune complex formation in Malians resulted in Fc-mediated inhibition of B cells via FcγIIB receptor, a major negative regulator of B cell activation and differentiation [19] or resulted in masking of important B cell epitopes [20]. Second, the Malian volunteers were exposed to malaria infections following vaccination, unlike their American counterparts. Data from a murine malaria model has shown that vaccination with MSP1_19_, another blood-stage antigen, followed by infection with *P. yoelii*, leads to apoptosis of MSP1-specific memory B cells [21]. Consequently, malaria antigen-specific memory B cells may undergo deletion, possibly through apoptosis, upon repeated infection with the parasite [21], [22]. Clearly, further study of the development of B cell memory responses to malaria antigens such as AMA1 is necessary.

### Generalizability

In this study, the AMA1-C1 malaria vaccine was well-tolerated and did not induce significant vaccine-related adverse events in malaria-exposed adults; however, any successful blood-stage malaria vaccine will be targeted primarily at infants and young children living in endemic areas since they bear the brunt of the morbidity and mortality due to this disease. Additional trials will therefore be required to establish safety in these age groups prior to initiation of larger efficacy studies. Furthermore, responses to vaccination with AMA1-C1 may be quite different in infants and young children living in endemic areas who have not been exposed to the antigen to the same extent as the semi-immune adults enrolled in the current study, especially considering the hypothesis that pre-existing anti-malarial antibodies might inhibit B cell activation as described above. Indeed, results from clinical trials of the RTS,S malaria vaccine indicate that humoral immune responses are lower in malaria-exposed adults than in malaria-exposed young children or malaria-naive adults [23]–[26].

Although there is considerable animal and human data supporting the development of AMA1 as a vaccine, a potential complication is the significant sequence polymorphism seen in strains isolated from different sites around the world. AMA1 has a minimum of 107 different haplotypes at the amino acid level (J Mu and LH Miller, unpublished data). Evidence that this polymorphism might affect vaccine efficacy comes from animal studies showing that rabbits immunized with one AMA1 haplotype produce antibodies that preferentially recognize homologous antigen, with reduced responses to heterologous AMA1 [9]. To overcome this potential obstacle to vaccine efficacy, AMA1-C1 incorporates both the 3D7 and FVO alleles of the protein in an attempt to elicit a broader immune response than that achievable by vaccination with either component alone. Whether recipients of the vaccine will be protected against parasites with polymorphic sequences (e.g., L32) remains to be determined.

In the current study, no association was seen between the significant increase in anti-AMA1 antibodies after the second vaccination with the 80 µg dose of AMA1-C1 and change in GIA activity. There are several potential reasons for this. First, pre-vaccination sera for most AMA1-C1 recipients displayed significant levels of growth inhibition, likely due to antibodies to a host of *P. falciparum* antigens induced by prior infections. The presence of antibodies to non-AMA1 antigens and the small number of participants in our study make interpretation of these GIA results difficult. However, preliminary evidence for the in vitro growth inhibition activity in these individuals being unrelated to antibody against AMA1 is the finding that in a subset of four post-vaccination serum samples with high levels of anti-AMA1 antibody, pre-incubation with AMA1 antigen failed to reverse growth inhibition.

### Overall evidence

In the first clinical trial of an AMA1 vaccine in an endemic area, the AMA1-C1 vaccine was safe when administered to malaria-exposed adults and, at the highest dose, was able to induce significant immune responses against the individual vaccine components as well as against a heterologous AMA1 allele. Improvements in the vaccine can clearly be made in order to elicit responses more likely to be sustained and protective, as individuals in the high-dose group who responded strongly after the first two vaccinations had minimal or no rise in AMA1-specific antibody levels after the third vaccination, and induction of antibody failed to translate into any significant increase in in vitro growth inhibition of the parasite. Different formulations of this vaccine–such as the addition of CpG oligodeoxynucleotideates or conjugate carriers [27], [28]–may overcome these limitations and result in induced antibody responses that are sufficient to impact parasite replication both in vitro and in vaccinated individuals. AMA1-C1 may nonetheless be a promising blood stage vaccine candidate, but further evidence is required from additional clinical trials, including studies conducted in children living in malaria-endemic areas.

## Supporting Information

Checklist S1 CONSORT Checklist
(0.11 MB PDF)

Protocol S1 Trial Protocol
(0.52 MB PDF)
