# Supplementary material for: Impact of a Plasmodium falciparum AMA1 Vaccine on Antibody Responses in Adult Malians
Source: PLoS One. 2007 Oct 17;2(10):e1045. doi: 10.1371/journal.pone.0001045 (PMC2013939; doi:10.1371/journal.pone.0001045)
Supplement: Checklist S1 — CONSORT Checklist (0.11 MB PDF) [file pone.0001045.s001.pdf]

CONSORT **Checklist of items to include when reporting a randomized trial**

PAPER SECTION And topic	Item	Description	Reported in Section
TITLE & ABSTRACT	1	How participants were allocated to interventions (*e.g.*, "random allocation", "randomized", or "randomly assigned").	Abstract-Design and Methods-Randomization
INTRODUCTION Background	2	Scientific background and explanation of rationale.	Introduction
METHODS Participants	3	Eligibility criteria for participants and the settings and locations where the data were collected.	Abstract-Setting/Participants and Methods-Study Site/Participants
Interventions	4	Precise details of the interventions intended for each group and how and when they were actually administered.	Abstract-Interventions and Methods-Interventions
Objectives	5	Specific objectives and hypotheses.	Abstract-Objectives and Methods-Objectives
Outcomes	6	Clearly defined primary and secondary outcome measures and, when applicable, any methods used to enhance the quality of measurements (*e.g.*, multiple observations, training of assessors).	Abstract-Outcome Measures and Methods-Outcomes
Sample size	7	How sample size was determined and, when applicable, explanation of any interim analyses and stopping rules.	Methods-Sample Size
Randomization -- Sequence generation	8	Method used to generate the random allocation sequence, including details of any restrictions (*e.g.*, blocking, stratification)	Methods-Randomization
Randomization -- Allocation concealment	9	Method used to implement the random allocation sequence (*e.g.*, numbered containers or central telephone), clarifying whether the sequence was concealed until interventions were assigned.	Methods-Randomization
Randomization -- Implementation	10	Who generated the allocation sequence, who enrolled participants, and who assigned participants to their groups.	Methods-Randomization
Blinding (masking)	11	Whether or not participants, those administering the interventions, and those assessing the outcomes were blinded to group assignment. If done, how the success of blinding was evaluated.	Methods-Blinding
Statistical methods	12	Statistical methods used to compare groups for primary outcome(s); Methods for additional analyses, such as subgroup analyses and adjusted analyses.	Methods-Statistical Methods
RESULTS Participant flow	13	Flow of participants through each stage (a diagram is strongly recommended). Specifically, for each group report the numbers of participants randomly assigned, receiving intended treatment, completing the study protocol, and analyzed for the primary outcome. Describe protocol deviations from study as planned, together with reasons.	Results-Participant Flow & Baseline Characteristics and Figure 1
Recruitment	14	Dates defining the periods of recruitment and follow-up.	Results-Participant Flow and Baseline Characteristics

Baseline data	15	Baseline demographic and clinical characteristics of each group.	Results-Participant Flow and Baseline Characteristics
Numbers analyzed	16	Number of participants (denominator) in each group included in each analysis and whether the analysis was by "intention-to-treat". State the results in absolute numbers when feasible (*e.g.*, 10/20, not 50%).	Results-Participant Flow and Baseline Characteristics
Outcomes and estimation	17	For each primary and secondary outcome, a summary of results for each group, and the estimated effect size and its precision (*e.g.*, 95% confidence interval).	Abstract-Results, Results-Safety, Results-IgG responses to AMA1-3D7 and AMA1-FVO, Results- IgG responses to AMA1-L32, and Results-Growth inhibition assay (GIA)
Ancillary analyses	18	Address multiplicity by reporting any other analyses performed, including subgroup analyses and adjusted analyses, indicating those pre-specified and those exploratory.	n/a
Adverse events	19	All important adverse events or side effects in each intervention group.	Abstract-Results and Results-Safety
DISCUSSION Interpretation	20	Interpretation of the results, taking into account study hypotheses, sources of potential bias or imprecision and the dangers associated with multiplicity of analyses and outcomes.	Discussion-Interpretation
Generalizability	21	Generalizability (external validity) of the trial findings.	Discussion-Generalizability
Overall evidence	22	General interpretation of the results in the context of current evidence.	Discussion-Overall evidence
