# Supplementary material for: Impact of a Plasmodium falciparum AMA1 Vaccine on Antibody Responses in Adult Malians
Source: PLoS One. 2007 Oct 17;2(10):e1045. doi: 10.1371/journal.pone.0001045 (PMC2013939; doi:10.1371/journal.pone.0001045)
Supplement: Protocol S1 — Trial Protocol (0.52 MB PDF) [file pone.0001045.s002.pdf]

DOUBLE-BLIND, RANDOMIZED, CONTROLLED, PHASE 1 STUDY OF THE SAFETY AND IMMUNOGENICITY OF AMA1-C1/ALHYDROGEL® VACCINE FOR *PLASMODIUM FALCIPARUM* MALARIA, IN SEMI-IMMUNE ADULTS IN DONEGUEBOUGOU, MALI

Version:	5.0 (March 28, 2005)
NIAID Protocol Number:	04-I-N175
FMPOS Protocol Number:	Malaria 002
FDA IND Number	BB-IND-10944
Ionizing Radiation:	None
Multi-institution:	Yes
Project Assurance:	FWA #0001769

Proposed start date:	April/May 2004
Proposed end date:	December 2005

<u>Sponsored by:</u>

Regulatory Compliance and Human Subjects Protection Branch (RCHSPB)
Office of Clinical Research (OCR), Office of the Director (OD)
National Institute of Allergy and Infectious Diseases (NIAID)
National Institutes of Health (NIH)

Team Roster

Principal Investigator:

Alassane Dicko, MD, MS
Malaria Research and Training Center (MRTC)
Department of the Epidemiology of Parasitic Diseases (DEAP)
Faculty of Medicine, Pharmacy and Dentistry (FMPOS), University of Bamako
BP 1805, Point G
Bamako, Mali
Tel/Fax: 223-222-8109
Email: adicko@mrtcbko.org

Co-Principal Investigator:

David Diemert, MD
Malaria Vaccine Development Branch (MVDB)
National Institute of Allergy and Infectious Diseases (NIAID)
5640 Fishers Lane, Twinbrook I
Rockville, MD 20852
(301) 443-3915
Email: ddiemert@niaid.nih.gov

Senior Co-Investigators:

Ogobara Doumbo, MD, PhD
MRTC/DEAP/FMPOS
University of Bamako, Mali

Dapa Aly Diallo, MD
MRTC/Dept. of Hematology/FMPOS

Clinical Investigators:

Mahamadou Aly Thera, MD, MPH
Issaka Sagara, MD, MSPH
Mahamadou S. Sissoko, MD, MSPH
Mohamed B. Niambélé, MD
Moussa Sogoba, MD
Beh Kamate, MD
Mahamadou H. Assadou, MD
Ousmane Guindo, MD
Moussa Konaré, MD
MRTC/DEAP/FMPOS
University of Bamako
Bamako, Mali

Elissa Malkin, DO, MPH
MVDB, NIAID/NIH
Rockville, Maryland

Clinical Investigators (cont.):

Christopher V. Plowe, MD, MPH
Center for Vaccine Development
University of Maryland
Baltimore, Maryland

Laboratory Investigators:

Mounirou Baby, PharmD
Amagana Dolo, PharmD, PhD
Boubacar Traoré, PharmD, PhD
Daniel Yalcouyé, PharmD
Mady Sissoko, PharmD
Mamadou Keita, PharmD
Abdou Salam Keita, MS
Mamadou Ba, MS
Modibo Daou, PharmD
MRTC/DEAP/FMPOS
University of Bamako
Bamako, Mali

Scientific Investigators:

Louis Miller, MD
Allan Saul, PhD
Carole Long, PhD
Greg Mullen, PhD
Siddhartha Mahanty, MD
MVDB, NIAID/NIH
Rockville, Maryland

NIAID Regulatory Affairs:

John Tierney, RN
OCR, OD
NIAID, NIH
6700-B Rockledge Drive, MSC 7609
Bethesda, MD 20892-7609
(301) 451-5136

Medical Monitors:

Aly Guindo, MD
Clinique Professeur Guindo
P.O. Box: E 2267, Bamako, Mali
Tel: (223) 222-2207
Fax: (223) 222-0195

Kalifa Sangaré, MD
Medecin Chef
Centre de Santé de Kati
Tel: (223) 227-2040

Participating Sites

Clinical Trial Site:

Donéguébougou Vaccine Center
MRTC/DEAP/FMPOS
Donéguébougou, Mali

Participating Laboratories

Clinical Laboratory:

MRTC/DEAP Clinical Laboratory
FMPOS, University of Bamako
BP 1805, Point G
Bamako, Mali

Immunology Laboratories:

Malaria Vaccine Development Branch
NIAID/NIH
5460 Fishers Lane, Twinbrook I
Rockville, MD 20852
Tel: (301) 435-3405

MRTC/DEAP Immunology Laboratory
FMPOS, University of Bamako,
BP 1805, Point G
Bamako, Mali

Table of Abbreviations

AE	Adverse Event
AMA1	Apical Membrane Antigen-1
AMA1-C1	Apical Membrane Antigen-1 Combination-1 Vaccine
CRF	Case Report Form
DEAP	Département d'Epidemiologie des Affections Parasitaires (Department of the Epidemiology of Parasitic Diseases)
DSMB	Data Safety and Monitoring Board
EIR	Entomologic Inoculation Rate
ELISA	Enzyme linked immunosorbent assay
ELISPOT	Enzyme linked ImmunoSPOT
EPI	Extended Program for Immunization
FDA	Food and Drug Administration
FMPOS	Faculté de Medicine, Pharmacie, et Odonto-Stomatology (Faculty of Medicine, Pharmacy, and Dentistry)
FWA	Federal Wide Assurance
GCP	Good Clinical Practices
GIA	Growth Inhibition Assay
HBsAg	Hepatitis B surface antigen
hCG	Human choriogonadotropin
HCV	Hepatitis C virus
ICH	International Conference on Harmonization
IRB	Institutional Review Board
Lf	Flocculation units
MMVDU	Mali Malaria Vaccine Development Unit
MRTC	Malaria Research and Training Center
MVDB	Malaria Vaccine Development Branch
NIAID	National Institute of Allergy and Infectious Diseases
NIH	National Institutes of Health
NMCP	National Malaria Control Program
PI	Principal Investigator
RCHSPB	Regulatory Compliance & Human Subjects Protection Branch
PNLP	Programme National de Lutte contre le Paludisme
SAE	Serious Adverse Event
SP	Sulfadoxine-pyrimethamine
TT	Tetanus toxoid vaccine
WHO	World Health Organization

Table of Contents

Team Roster 2
Table of Abbreviations 5
Protocol Summary 11
1.0 Introduction 13
1.1 Background 13
1.1.1 Malaria as a Public Health Problem 13
1.1.2 Disease burden in Mali 13
1.2 The AMA1 Protein 14
1.2.1 AMA1-C1 Vaccine 15
1.3 AMA1-C1 Vaccine Description 16
1.3.1 AMA1 16
1.3.2 Alhydrogel® 16
1.3.3 Vaccine Production & Formulation 17
1.4 Rationale for using AMA1 as a Malaria Vaccine Antigen 17
1.4.1 Animal Experience with AMA1 Homologue Vaccines 17
1.4.2 Preclinical Experience with AMA1-C1 18
1.4.3 Clinical Experience with AMA1-C1/Alhydrogel® 21
1.4.4 Previous Human Experience with AMA1 3D7/Montanide ISA 720 23
1.4.5 Clinical Experience with Aluminum-Based Adjuvants 24
1.5 Clinical Development Plan 24
1.5.1 Participation of Children 25
1.6 Comparator Vaccine 25
1.6.1 Rationale for use of a comparator vaccine 25
1.6.2 Rationale for use of the Hepatitis B vaccine as a comparator 26
2.0 Objectives 27
2.1 Primary Objective 27
2.2 Secondary Objectives 27
3.0 Study Site 27
4.0 Study Design 29
4.1 Overall Design 29
4.2 Sample Size and Estimated Duration of Study 31
4.3 Timing of Third Vaccination (Day 360) 31
4.4 Group Allocation 32
4.5 Blinding 32
5.0 Selection and Enrollment of Volunteers 33
5.1 Inclusion Criteria 33
5.2 Exclusion Criteria 33
5.2.1 Rationale for Use of Clinical Assessments of Immunosuppression 34
5.3 Treatments That Could Potentially Interfere with Vaccine-Induced Immunity 35
5.4 Contraindications to Vaccination 35
5.5 Indications for Deferral of Vaccination 35
5.6 Subject Withdrawal Criteria 36
6.0 Vaccine Preparation 36
6.1 Supplies 36
6.2 Vaccine Storage 37
6.3 Vaccine Accountability 37
6.4 Disposition of Used/Unused Supplies 37
7.0 Study Procedures 37
7.1 Community Consent 38

7.2 Individual Recruitment and Informed Consent 38
7.3 Randomization Process 39
7.4 Enrollment 40
7.5 Immunization Procedure 40
7.6 Clinical Monitoring and Evaluation 40
7.7 Laboratory Testing 45
7.8 Immunologic Testing 45
7.8.1 Antibody Assay 45
7.8.2 Growth Inhibition Assay 46
7.8.3 B cell analysis 46
7.8.4 Plasmodium falciparum genotyping 46
8.0 Adverse Events Monitoring and Reporting 47
8.1 Definitions 47
8.1.1 Adverse Event 47
8.1.2 Serious Adverse Event (SAE) 47
8.2 Assessment of Adverse Events 47
8.2.1 Identification of AEs 47
8.2.2 Determination of Severity 48
8.2.3 Association with Receipt of the Study Vaccine 49
8.3 Adverse Event Reporting 50
8.4 Adverse Event Monitoring 51
8.4.1 Medical Monitor 51
8.4.2 Data Safety and Monitoring Board (DSMB) 51
8.5 Criteria for Placing the Study on Hold 52
8.6 Breaking the Study Blind 52
9.0 Data Collection and Monitoring 53
9.1 Source Documentation 53
9.2 Study Documentation 53
9.2.1 Study Reports 54
9.3 Retention of Records 54
9.4 Protocol Revisions 54
9.5 Clinical Investigator's Brochure 54
9.6 Study Monitoring 55
10.0 Statistical Considerations 55
10.1 General Design 55
10.1.1 Description of the Statistical Methods to Be Employed 55
10.1.2 Safety 57
10.1.3 Immunogenicity Analysis 57
10.2 Sample Size 57
11.0 Protection of Human Subjects 58
11.1 Institutional Review Board/Ethics Committee 58
11.2 Informed Consent 58
11.3 Risks 58
11.3.1 Venipuncture 59
11.3.2 Immunization with AMA1-C1/Alhydrogel® 59
11.3.3 Immunization with Recombivax HB® 59
11.4 Precautions taken to Minimize Risks 59
11.4.1 Immunization 59
11.4.2 Protection of Study Staff 60
11.5 Benefits 60
11.6 Confidentiality 60

11.7 Compensation 61
12.0 References 61
Appendix A – Summary of Preclinical Studies for AMA1-C1 65
Appendix B – Map of Donéguébougou Village 67
Appendix C – Malaria Vaccine Consent Comprehension Exam 68
Appendix D – Schedule of Visits 69
Appendix D – Schedule of Visits (continued) 70
Appendix E – Toxicity Table for Grading Laboratory Adverse Events 71

List of Tables

Table 1 In vitro Growth Inhibition[1] of *P. falciparum* FVO and 3D7 Merozoites When Incubated with Serial Dilutions of Rabbit Sera Raised against AMA1 FVO, AMA1 3D7, or a Combination of Both Antigens (AMA1-C1)[2] 20
Table 2 Summary of Local Adverse Events Related to Vaccination from US AMA1-C1/Alhydrogel® Clinical Trial 21
Table 3 Summary of Solicited Systemic Adverse Events from US AMA1-C1/Alhydrogel® Clinical Trial 22
Table 4 Anti-AMA1 Specific Antibody Units in Humans Immunized with AMA1-C1/Alhydrogel®, as Determined by ELISA, 14 Days Following Administration of the Second Immunization 23
Table 5 The 2003 Donéguébougou population distribution, by age group 28
Table 6 Dose Escalation Schedule 31
Table 7 Assessment of Adverse Event Intensity 49

List of Figures

Figure 1 Comparison of Day 42 Anti-AMA1 Specific Antibody Levels[1] in Rabbits (n=5) Immunized with AMA1-C1/Alhydrogel® 19
Figure 2 Day 42 Anti-AMA1 Specific Antibody Levels[1] in Mice (n=48) Immunized with AMA1-C1/Alhydrogel® 19

STATEMENT OF THE DIRECTOR OF THE MALARIA RESEARCH AND TRAINING CENTER OF THE DEPARTMENT OF THE EPIDEMIOLOGY OF PARASITIC DISEASES, FACULTY OF MEDICINE, PHARMACY, AND DENTISTRY, UNIVERSITY OF BAMAKO, MALI:

I, the undersigned, have reviewed this protocol and have approved it. I will ensure that the clinical study as described will adhere to the principles of the ICH/cGCP as well as all applicable regulatory requirements. I have read and understood the contents of the Investigator's Brochure provided by the Malaria Vaccine Development Branch of the US National Institute of Allergy and Infectious Diseases.

Ogobara Doumbo, MD, PhD
Director of the MRTC ____________________________________
Signature

____
Date

PRINCIPAL INVESTIGATOR'S STATEMENT:

I, the undersigned, have reviewed this protocol, including Appendices, and will conduct the clinical study as described and will adhere to the principles of the ICH/cGCP as well as all applicable regulatory requirements. I have read and understood the contents of the Investigator's Brochure provided by the Malaria Vaccine Development Branch of the US National Institute of Allergy and Infectious Diseases.

Alassane Dicko, MD, MS ____________________________________
Signature

____
Date

Protocol Summary

Title Double-blind, randomized, controlled Phase 1 Study of the Safety and Immunogenicity of AMA1-C1/Alhydrogel® Vaccine for *Plasmodium falciparum* Malaria, in Semi-immune Adults in Donéguébougou, Mali

Study Population Healthy, malaria-exposed male and non-pregnant female volunteers aged 18-45 years, inclusive.

Rationale Apical membrane antigen-1 (AMA1) is a surface protein expressed during the asexual blood stage of *P. falciparum*. It is produced as an 83-kDa polypeptide by mature schizonts in infected erythrocytes. In a clinical study in malaria-unexposed adults in the USA, AMA1-C1/Alhydrogel® was safe and immunogenic. This study will evaluate its safety and immunogenicity in malaria-exposed individuals living in an area of seasonal malaria transmission.

Study Design:

- Double-blind, randomized, controlled Phase 1 clinical trial.
- Study center: Donéguébougou, Mali.
- Number of volunteers: 54 in three groups of 18, randomized 2:1 to receive either AMA1-C1/Alhydrogel® or Recombivax HB® Hepatitis B vaccine.
- Study duration: 83 weeks; each volunteer will be followed for a total of 78 weeks.
- Immunization schedule: Study days 0, 28 and 360.
- Route: IM in the deltoid muscle.
- Dose of AMA1-C1: 5, 20 and 80 µg for the first, second and third dose cohort, respectively.
- Dose of Alhydrogel®: 800 µg for each dose of AMA1-C1.

Immunization Schedule:

Cohort	Total Volunteers	Immunization Schedule		
		Day 0	Day 28	Day 360
1	18	A(12) + D(6)	A(12) + D(6)	A(12) + D(6)
2	18	B(12) + D(6)	B(12) + D(6)	B(12) + D(6)
3	18	C(12) + D(6)	C(12) + D(6)	C(12) + D(6)
Total	54	A: 5 µg AMA1-C1/Alhydrogel®<br>B: 20 µg AMA1-C1/Alhydrogel®<br>C: 80 µg AMA1-C1/Alhydrogel®<br>D: Recombivax HB®		

Objectives:

Primary

1. To estimate the frequency of vaccine-related AEs, graded by severity, for each dose.

Secondary

1. To determine the dose of AMA1-C1 that generates the highest antibody concentration at Day 42, as judged by enzyme-linked immunosorbent assay (ELISA).
2. To assess and compare the duration of antibody response to AMA1 FVO and 3D7.
3. To measure the inhibition of parasite growth as measured by the in vitro GIA to FVO and 3D7.
4. To determine the relationship between anti-AMA1 antibody concentration, as judged by ELISA, and degree of in vitro growth inhibition of *P. falciparum*.
5. To determine the relative specificity of the antibodies to a range of AMA1 serotypes in addition to FVO and 3D7, as judged by ELISA, and growth inhibition on a select panel of parasites with typed AMA1.
6. To perform exploratory studies of B cell populations both before and after immunization.

Product Description:

The AMA1-C1 vaccine preparations to be studied contain an equal mixture of AMA1 from two different clones of *Plasmodium falciparum* (FVO and 3D7), both produced separately as recombinant proteins expressed by *Pichia pastoris* (AMA1 FVO and AMA1 3D7). Purified AMA1 FVO and AMA1 3D7 were subsequently mixed and adsorbed onto aluminum hydroxide gel (Alhydrogel®).

The comparator vaccine is the licensed Recombivax HB® Hepatitis B vaccine (Merck & Co.), containing recombinant Hepatitis B surface protein expressed by *Saccharomyces cerevisiae*, adsorbed to amorphous aluminum hydroxyphosphate sulfate.

1.0 INTRODUCTION

1.1 Background

1.1.1 Malaria as a Public Health Problem

As reported by the World Health Organization in 1997, the worldwide incidence of malaria is approximately 300–500 million clinical cases annually, with between 2-3 million deaths per year attributed to malaria alone or in combination with other diseases [1]. Most of the mortality occurs among children under 5 years of age in sub-Saharan Africa. Of the four species of malaria that infect humans, *Plasmodium falciparum* is responsible for the majority of these deaths. Mounting drug resistance of the malaria parasite, as well as widespread resistance of mosquitoes to insecticides make these control strategies increasingly inadequate. A vaccine that would reduce both mortality and morbidity secondary to *P. falciparum* infection would be a valuable new resource in the fight against this disease.

P. falciparum has a complex life cycle. Sporozoites – the infectious stage of the parasite – are transmitted to humans through the saliva of infected mosquitoes while they are taking a blood meal. Sporozoites travel through the bloodstream to the liver, where they invade hepatocytes and then undergo a series of divisions to develop into merozoites. Six to 10 days after invasion, the hepatocytes rupture, releasing thousands of merozoites into the bloodstream. These merozoites invade erythrocytes where they multiply, and after 2 days, release progeny merozoites, which subsequently invade new erythrocytes to continue the asexual blood-stage cycle. Clinical symptoms in humans are due to this asexual blood stage of the parasite's life cycle. A small percentage of merozoites do not multiply after invading erythrocytes, but instead differentiate into gametocytes. These gametocytes are ingested by a mosquito during a subsequent blood meal and undergo sexual reproduction in the mosquito midgut, producing a zygote. The zygote matures and releases sporozoites that migrate to the mosquito's salivary glands, thus completing the life cycle.

1.1.2 Disease burden in Mali

In Mali, malaria is the leading cause of mortality (13%) and morbidity (15.6%) in the general population [2]. However, there are large geographical differences in malaria infection rates and in disease prevalence and incidence, depending on a variety of factors such as the climate (e.g., the amount of rainfall and the duration of rainy season), the type of agriculture practiced (e.g., rice versus millet production), and access and utilization of health care and protective measures (e.g., insecticide-treated bednets). Malaria transmission in Mali is highly seasonal, occurring primarily during the rainy season which lasts between 3 to 6 months, depending on the geographic location within Mali.

The prevalence of *Plasmodium* infection (as determined by microscopy) often exceeds 70% in children aged 2 to 9 years during the rainy season. According to epidemiological data collected by the Département d'Epidémiologie des Affections Parasitaires (DEAP) of the University of Bamako, the incidence of clinical malaria varies between 1.5 to 2 episodes per child per year, with some children experiencing up to 5 episodes of clinical

malaria per year. Furthermore, the National Malaria Control Program (NMCP) of Mali reports that malaria fever represents 34% of all outpatient consultations in the country. Severe malaria accounts for 15% of hospitalizations in children between the ages of 0 and 14 years in the capital city of Bamako, with case fatality rates of approximately 17% at the National Pediatric Hospital in Bamako, versus 25% countrywide [3-6]. The most common presentation of severe malaria is cerebral malaria (61 to 84% of cases), whereas severe anemia is the cause of 8 to 30% of cases [4, 5].

1.2 The AMA1 Protein

Several *P. falciparum* antigens have been identified as potential vaccine components [7]. Proteins expressed by *P. falciparum* are generally specific to one stage of the parasite's life cycle. The apical membrane antigen-1 (AMA1) is a surface protein expressed during the asexual blood stage of *P. falciparum*. AMA1 is produced as an 83 kDa polypeptide by mature schizonts in infected erythrocytes [8], and localizes in the microneme, an apical secretory organelle of the merozoite containing ligands for binding red cell receptors [9]. The protein is processed to a 66 kDa protein that is subsequently exported to the merozoite surface at around the time of rupture of the schizont-infected erythrocyte [10]. Although its exact function remains undetermined, these observations suggest that it performs a role during merozoite invasion of erythrocytes. Further evidence supporting this comes from studies with monoclonal antibodies to primate and murine plasmodia AMA1 that exhibit in vitro inhibition of parasite invasion of erythrocytes [12, 13]. This invasion inhibition is not a result of parasite agglutination by antibody, as it can be demonstrated that even the Fab fragments of these monoclonal antibodies block such invasion [14].

P. falciparum AMA1 consists of a signal sequence, a large extracellular domain (ectodomain), a transmembrane domain, and a short cytoplasmic tail. In comparison to nonhuman primate and mouse malaria parasites, *P. falciparum* AMA1 has a 45 amino acid extension after the signal sequence that is missing from all other *Plasmodium* species except the *P. falciparum*-like simian parasite *P. reichenowi*. Comparisons between all of the known amino acid sequences of AMA1 homologues indicate greater than 50% sequence identity, with 16 cysteine residues conserved in all sequences [10, 15, 16]. All of the cysteines are found in the ectodomain of the molecule, which is stabilized by eight intramolecular disulfide bonds [17].

AMA1 lacks the sequence repeats observed in other malaria antigens such as the merozoite surface antigens MSP-1 and MSP-2. However, sequence polymorphism resulting from point mutations is observed among alleles of the single copy AMA1 gene in *P. falciparum* [11, 15, 18]. Escalante et al. compared sequences from a total of 44 *P. falciparum* isolates from Kenya, India, Thailand, and Venezuela and observed polymorphism at 118 out of 622 amino acids [19]. No insertion/deletion mutations were observed, although approximately 70% of the mutations in the gene encoding AMA1 result in nonsynonymous substitutions, suggesting positive natural selection. Evidence from this study and previous ones indicate that mutations are predominantly clustered within three regions of the ectodomain that are defined by the eight disulfide bonds and affect both B and T-cell epitopes [15, 20].

Seroepidemiologic studies conducted in West Africa and western Kenya demonstrate that natural antibody responses to AMA1 are widespread. Thomas et al. conducted a cross-sectional study of antibody responses to recombinant AMA1 in children ages 2-9 in Guinea-Bissau, an area of moderate endemnicity, and in people ages 2-86 in Senegal, an area of holoendemic transmission [21]. Overall, a very high prevalence (94%-100%) of naturally acquired serum IgG responses to AMA1 were recorded. Although significant age-dependent changes in the serum antibody levels to this antigen were found among children in Guinea-Bissau, no such correlation was observed among children in Senegal, possibly because high antibody levels develop at an earlier age in areas of more intense malaria transmission. Although not designed to detect such a relationship, this study did not find any correlation between antibody response and protection from parasitemia or fever episodes.

A second study was performed by Udhayakumar et al. in western Kenya, an area of holoendemic, perennial transmission, to determine the development and maintenance of proliferative and antibody responses to AMA1 epitopes [22]. This study showed that proliferative responses and serum antibody responses to B epitopes reached their peak prevalence and magnitude in 5-9-year-olds for most epitopes, an age by which most children have developed clinical immunity to malaria in this area. Serum antibody levels remained stable throughout a single transmission season, unlike T-cell proliferative responses, which were transient and short-lived.

Preliminary studies in Donéguébougou, Mali, demonstrate that natural antibodies exist in a large proportion of individuals (>90%) in this area of intense, seasonal, transmission. In a cross-sectional study of 200 individuals aged 6 months to 45 years conducted in 2002 and 2003, median anti-AMA1 antibody levels reached approximately 1000 units/mL at the peak of malaria transmission, although significant variation was seen between different age groups [40].

Not only have natural antibodies to AMA1 been demonstrated in people living in malaria-endemic areas, these antibodies have also been shown to inhibit the in vitro growth of *P. falciparum* [23]. In this study, human anti-AMA1 IgG was affinity purified from a pool of plasma obtained from Papua New Guinean blood donors who had previously been found to have high titers of antibodies to a variety of *P. falciparum* asexual blood-stage antigens. When tested in the in vitro merozoite invasion assay, the affinity-purified human anti-AMA1 antibodies inhibited various strains of *P. falciparum* in a manner similar to that observed with rabbit IgG raised to refolded *P. falciparum* AMA1 3D7. This inhibition was both dose-dependent and strain-specific. From this evidence, it is reasonable to postulate that boosting the natural antibody response to AMA1 through vaccination may protect an individual from illness due to the asexual blood stage of *P. falciparum* infection.

1.2.1 AMA1-C1 Vaccine

The AMA1-C1 vaccine being tested in this study contains an equal mixture of the correctly folded ectodomain portion of recombinant AMA1 from two different clones of

P. falciparum: the FVO and 3D7 clones. The proteins AMA1 FVO and AMA1 3D7 were expressed separately as secreted recombinant proteins in *Pichia pastoris*, purified, and then combined in equal amounts by mass. A combination vaccine was chosen because of concerns about parasite polymorphism and evidence for strain-specific protection as outlined above. These two strains were chosen because their sequences are sufficiently different, when comparing all sequenced *P. falciparum* clones [19]. The intent is to provide a broad range of protection against the different strains of the parasite occurring in the field. Although relatively different among all known AMA1 sequences, the two recombinant proteins in the vaccine remain more than 95% homologous, the cysteine residues are in identical positions, and both are produced in the same expression system (*P. pastoris*). Additionally, the fermentation and purification of the two proteins are performed by procedurally similar batch production records.

Further evidence that combining two forms of AMA1 will help overcome the polymorphism of the AMA1 allele and thus provide broader protection in the field comes from studies that the Malaria Vaccine Development Branch (MVDB) has completed in rabbits. When immunized with a combination of recombinant AMA1 FVO and AMA1 3D7, titers of antibody to each individual antigen were similar to the titers obtained in rabbits immunized with each antigen separately. Furthermore, antibody from those animals immunized with the combination vaccine (AMA1-C1), when mixed with both 3D7 and FVO parasites in vitro, inhibited parasite growth equally (see **Section 1.4.2** in this protocol for more detailed description of results). When sera from rabbits immunized with only a single form of AMA1 were tested against the heterologous parasite, considerably less inhibition was seen than against the homologous parasite.

1.3 AMA1-C1 Vaccine Description

1.3.1 AMA1

Both recombinant AMA1 FVO and AMA1 3D7 AMA1 3D7 are highly purified 62 kDa proteins that correspond to the ectodomain of *P. falciparum* (FVO) AMA1 and *P. falciparum* (3D7) AMA1, respectively. Both forms of AMA1 consist of amino acids 25 through 545 of the published sequences of each clone's AMA1 (GenBank accession number AJ277646 for FVO and accession number U65407 for 3D7). AMA1 FVO and AMA1 3D7 each consist of the ectodomain of the mature protein found in parasites with the addition of a 6-histidine C-terminal tag to allow purification of the protein. The proteins AMA1 FVO and AMA1 3D7 were expressed separately as secreted recombinant proteins in *P. pastoris* and purified by a combination of affinity, ionic, hydrophobic, and gel filtration chromatography.

1.3.2 Alhydrogel®

Aluminum hydroxide gel (HCI Biosector, Denmark) has been extensively used as an adjuvant in many licensed human vaccines. Aluminum-containing adjuvants are in routine human use and contained in many licensed human vaccines.

1.3.3 Vaccine Production & Formulation

The synthetic AMA1 gene sequences were subcloned into the *P. pastoris* expression plasmid, pPIC9K (Invitrogen Corporation, Carlsbad, California), which encodes a pre-prosecretory α-factor sequence that is cleaved by the yeast enzyme KEX2 during protein maturation. AMA1 FVO and AMA1 3D7 were purified from the fermentation supernatant using a combination of affinity, ionic, hydrophobic, and gel filtration chromatography. The purification process was designed to separate full-length product from degraded material as well as non-product-related contaminants. AMA1 FVO and AMA1 3D7 bulk antigens (drug substances) were both manufactured at the WRAIR Bioproduction Facility (Silver Spring, Maryland) according to cGMP. Immediately prior to formulation, equal weights of AMA1 FVO and AMA1 3D7 were mixed and then bound to Alhydrogel®. AMA1-C1 refers to the mixture of AMA1 FVO and AMA1 3D7. The AMA1-C1/Alhydrogel® vaccine refers to AMA1-C1 formulated on Alhydrogel®.

1.4 Rationale for using AMA1 as a Malaria Vaccine Antigen

1.4.1 Animal Experience with AMA1 Homologue Vaccines

Vaccination with both purified native and recombinant AMA1 has been shown to protect against malaria infection in both rodent and simian models. Immunization with the affinity-purified *P. knowlesi* AMA1 homologue was first demonstrated by Deans et al. to protect rhesus monkeys against homologous parasite challenge; protection appeared to correlate with anti-AMA1-specific antibody titer [24]. Similarly, Collins et al. were able to obtain partial protection against *P. fragile* infection in *Saimiri* monkeys following immunization with recombinant *P. fragile* AMA1 [25], once again correlated to antibody level. In this study, vaccination with just the ectodomain portion of the recombinant protein gave similar protection as vaccination with the full-length protein, indicating that a vaccine consisting of only this portion of the molecule confers adequate protection. Even more encouraging, monkeys from this study were subsequently challenged with *P. falciparum* after having been vaccinated and challenged with *P. fragile*; none of these animals developed parasitemia.

Mice immunized with the *P. chabaudi adami* (DS) AMA1 ectodomain expressed in *Escherichia coli* and refolded in vitro have displayed high levels of protection against homologous challenge in four separate studies [26-29]. Furthermore, passive immunization of AMA1 specific polyclonal antibodies raised by immunizing rabbits with *E. coli* expressed and refolded *P. chabaudi adami* AMA1 into *P. chabaudi* infected mice prevented lethal parasitemias with homologous parasite [27, 29]. Notably, in this murine model, protection was limited to the homologous parasite: when mice immunized with recombinant *P. chabaudi adami* (DS) AMA1 were challenged with *P. chabaudi adami* (556KA), no protection was observed [29]. Most recently, purified native *P. yoelii yoelii* YM AMA1 has been shown to protect against homologous parasite challenge [30]. Protection once again correlated with antibody titer.

Several conclusions can be drawn from the accumulated animal data of vaccination trials with both purified native and recombinant AMA1. First, the protective efficacy of this molecule requires its native confirmation. Studies comparing reduced versus correctly

folded recombinant *P. chabaudi* AMA1 [27, 29] and affinity-purified *P. knowlesi* AMA1 [24] demonstrated protection from parasite challenge only with the correctly folded protein, despite similar anti-AMA1 antibody levels in the two groups. Furthermore, passive transfer of polyclonal anti-AMA1 antibodies raised in rabbits immunized with recombinant *P. chabaudi* AMA1 protected mice against homologous parasite challenge only when antibodies were raised against correctly folded AMA1 [27, 29]. Second, the Collins et al. finding that immunization with just the ectodomain portion of the AMA1 molecule provides protection equal to that obtained with the entire molecule has been replicated in several additional studies in both mice and nonhuman primates [28, 29, 31].

Finally, animal data from other investigators' vaccine trials as well as the preclinical experience with the AMA1-C1 vaccine (see **Section 1.4.2** in this protocol) indicate that the observed polymorphism in AMA1 may impact on the ability of a vaccine based on a single sequence of AMA1 to protect against heterologous parasite infection. As already described, immunization of mice with recombinant *P. chabaudi adami* (DS) AMA1 protected against challenge with homologous but not heterologous parasite challenge [29]. However, in another study by Hodder et al., anti-AMA1 antibodies raised in rabbits immunized with recombinant *P. falciparum* 3D7 were tested against the 3D7, D10, and HB3 *P. falciparum* clones in erythrocyte invasion inhibition assays [23]. Significant invasion inhibition was seen with the homologous and D10 parasites although less (but still greater than 50% inhibition) was seen against the HB3 clone. Of the AMA1 alleles sequenced, HB3 is one of the most distant from the 3D7 sequence, with 23 amino acid differences in the ectodomain, in contrast with only 8 amino acid differences between the 3D7 and D10 sequences. In the same study, affinity-purified anti-AMA1 antibodies from residents of Papua New Guinea were tested against the same three parasite clones in the in vitro merozoite invasion assay. Similar results were obtained in humans as with the rabbit IgG raised to the refolded *P. falciparum* AMA1 3D7 ectodomain. HB3 exhibited less inhibition as compared to the homologous 3D7 or D10 parasite lines. These studies suggest that protection may be variable depending on the sequence similarity between the infecting clone and that used in a vaccine.

1.4.2 Preclinical Experience with AMA1-C1

The AMA1-C1 vaccine has been tested in mice, rats, rabbits, guinea pigs, and both New and Old World monkeys. A total of 17 individual preclinical trials have been, or are being, conducted to assess the safety, immunogenicity, and toxicology of the recombinant 3D7 and FVO AMA1 proteins when administered with several different adjuvants (see **Appendix A** for a tabular summary of the preclinical trials). These studies have demonstrated the safety of AMA1 3D7 and AMA1 FVO when administered either separately or in combination as the AMA1-C1 vaccine, when adjuvanted with Alhydrogel®. Immunogenicity data from BALB/c mice, *Rattus norvegicus* rats, guinea pigs, New Zealand White rabbits, *Aotus vociferans* monkeys, and rhesus monkeys have demonstrated that all six animal species develop significant anti-AMA1 antibody responses. Mouse, rabbit, guinea pig, and monkey studies have compared AMA1 formulated with Alhydrogel®, Montanide ISA 720, QS21 + Polysorbate 80, MF59, Alhydrogel® + RC529, Alhydrogel® + CpG 10105, and Alhydrogel® + CpG 5470. Data

from eight mice studies (combined n=968), four rabbit studies (combined n=79), one rat study (n=24), and one guinea pig study (n=45) have demonstrated significant antibody responses to both AMA1 FVO and AMA1 3D7 (see **Figures 1** and **2)**. Additionally, in a trial of 30 rhesus monkeys, significant antibody production was elicited to both AMA13D7 and AMA1 FVO when administered separately or in combination.

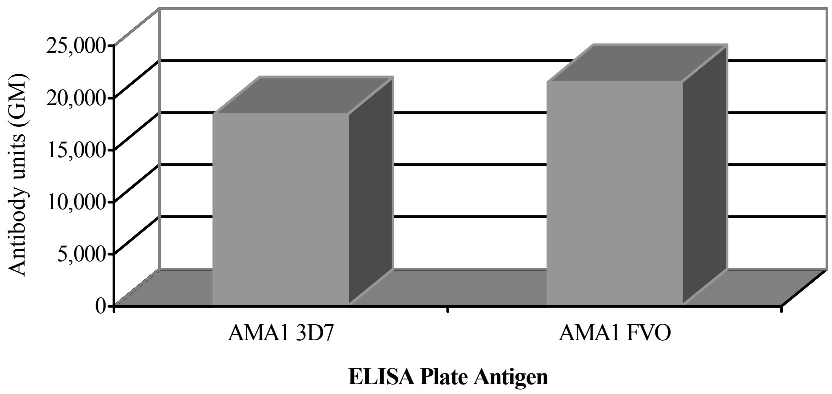

[1]Expressed as the geometric means (GM)

Figure 1 Comparison of Day 42 Anti-AMA1 Specific Antibody Levels[1] in Rabbits (n=5) Immunized with AMA1-C1/Alhydrogel®

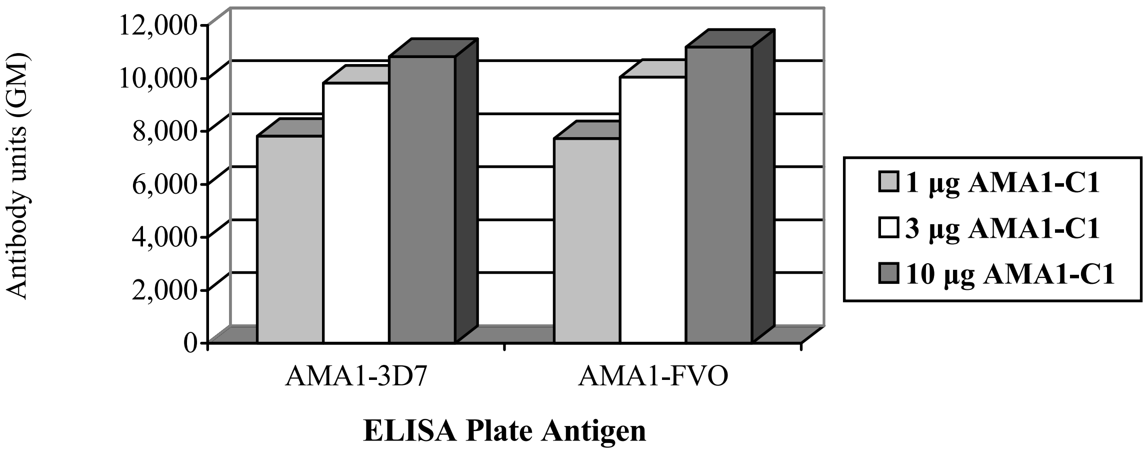

[1]Expressed as the geometric means (GM)

Figure 2 Day 42 Anti-AMA1 Specific Antibody Levels[1] in Mice (n=48) Immunized with AMA1-C1/Alhydrogel®

Several observations can be drawn from the results of the three rabbit immunogenicity studies that have been completed. First, administering either AMA1 FVO or AMA1 3D7 to rabbits elicited a significant antibody response to the homologous protein, whereas a markedly reduced antibody response was produced to heterologous protein. However, when administered in combination (i.e., AMA1 FVO + AMA1 3D7), significant and equivalent antibody responses to both proteins were observed. Similarly, when tested in the in vitro merozoite growth inhibition assay (GIA), these antibodies significantly inhibited growth of homologous parasite, but inhibited heterologous parasite growth to a

lesser degree (see **Table 1**). In contrast, anti-AMA1 antibody elicited by administering the combination of AMA1 FVO and AMA1 3D7 to rabbits inhibited the in vitro growth of both *P. falciparum* FVO and *P. falciparum* 3D7 to a similar degree. Thus, protection in rabbits varied depending on the relative homology between the form used to immunize and that used in the GIA, although this variable protection was overcome by immunizing with a combination of the two proteins.

Table 1 In vitro Growth Inhibition[1] of *P. falciparum* FVO and 3D7 Merozoites When Incubated with Serial Dilutions of Rabbit Sera Raised against AMA1 FVO, AMA1 3D7, or a Combination of Both Antigens (AMA1-C1)[2]

Group	*P. falciparum* clone				
	FVO	3D7	D10	M24	HB3
AMA1 FVO	71.4%	19.8%	47.3%	29.4%	45.0%
AMA1 3D7	14.8%	81.5%	95.5%	16.0%	33.9%
AMA1-C1	66.0%	64.6%	92.5%	28.5%	48.9%

[1]Groups of four rabbits were immunized with AMA1 FVO, AMA1 3D7, and an equimolar amount of the two proteins. Sera were collected 2 weeks after the second immunization, and the anti-AMA1 antibodies were assayed for their ability to prevent invasion of red blood cells by both homologous and heterologous *P. falciparum* parasites in a GIA.
[2]Results are expressed as mean % growth inhibition.

No clinical abnormalities (injection-site reactions and general health observations) were observed in any of the 664 mice that received AMA1-C1/Alhydrogel®. Of the 55 rabbits in the three immunogenicity studies, 5 received AMA1-C1/Alhydrogel®: these rabbits remained in good general health throughout the study. Safety data from the 30-monkey rhesus study indicate that among the 6 monkeys vaccinated with AMA1-C1/Alhydrogel®, there were no significant injection-site reactions or unexpected changes in weight.

In addition, a formal toxicology study was performed in New Zealand White rabbits to assess clinical-grade AMA1-C1/Alhydrogel®. In this study, 24 rabbits (12 male and 12 female) received either Alhydrogel® alone or 80 µg AMA1-C1/Alhydrogel®. The immunizations were administered as 0.5 mL intramuscular (IM) injections on Days 0, 28, 49, and 70. No significant injection-site reactions, weight changes, or alterations in food consumption were observed during the course of this study. Significant antibody responses were observed following administration of 80 µg of AMA1-C1/Alhydrogel®.

Finally, a vaccination-challenge study was performed in *Aotus vociferans* monkeys, using preclinical-grade AMA1 FVO with Complete Freund's adjuvant and Incomplete Freund's adjuvant [36]. In this study, 100 µg of AMA1 FVO was administered subcutaneously (SC) either alone or in combination with another asexual blood-stage antigen, MSP1-42. Of the six animals vaccinated with AMA1 FVO alone, two were completely protected from an intravenous challenge with homologous parasites and had no parasitemia; two developed very low-grade parasitemias (< 0.75%); and one animal had a marked delay in development of parasitemia. Protection correlated significantly with anti-AMA1 antibody titer.

1.4.3 Clinical Experience with AMA1-C1/Alhydrogel®

The AMA1-C1/Alhydrogel® malaria vaccine has been tested in one human clinical trial to date. This Phase 1 trial began in July 2003 in the United States and is currently in progress. A total of 30 healthy, malaria-unexposed adult volunteers have been enrolled in an open-label dose-escalation study testing the same three doses of AMA1-C1/Alhydrogel® as in the study described in this protocol (5, 20, and 80 µg of AMA1-C1). Thus far, all volunteers have received two doses of vaccine (given on Study Days 0 and 28), and all have been followed until at least Day 42 (2 weeks after receiving the second dose). A third and final dose will be given on study Day 180.

The cumulative safety data from this trial is presented in **Tables 2** and **3**; the AMA1-C1-specific induced antibody results are listed in **Table 4**. In brief, this vaccine has been shown to be safe and immunogenic in healthy, malaria-unexposed adults, with injection-site tenderness (mild to moderate) being the most common vaccine-related adverse event. No vaccine-related SAEs nor severe adverse events have been observed to date.

Table 2 Summary of Local Adverse Events Related to Vaccination from US AMA1-C1/Alhydrogel® Clinical Trial

	**Vaccination #1**				**Vaccination #2**			
**5 µg**	**Total**	**Mild**	**Moderate**	**Severe**	**Total**	**Mild**	**Moderate**	**Severe**
Tenderness	6/10	1	5	0	6/10	1	5	0
Erythema	1/10	1	0	0	4/10	4	0	0
Induration	3/10	3	0	0	4/10	4	0	0
**20 µg**	**Total**	**Mild**	**Moderate**	**Severe**	**Total**	**Mild**	**Moderate**	**Severe**
Tenderness	3/10	2	1	0	2/9	1	1	0
Erythema	2/10	2	0	0	4/9	4	0	0
Induration	4/10	4	0	0	3/9	3	0	0
**80 µg**	**Total**	**Mild**	**Moderate**	**Severe**	**Total**	**Mild**	**Moderate**	**Severe**
Tenderness	6/10	4	2	0	3/9	2	1	0
Erythema	0/10	0	0	0	1/9	1	0	0
Induration	3/10	2	1	0	3/9	3	0	0

Table 3 Summary of Solicited Systemic Adverse Events from US AMA1-C1/Alhydrogel® Clinical Trial

	Vaccination #1				Vaccination #2			
**5 μg**	**Total**	**Mild**	**Moderate**	**Severe**	**Total**	**Mild**	**Moderate**	**Severe**
Fever	0/10	0	0	0	0/10	0	0	0
Headache	1/10	1	0	0	0/10	0	0	0
Nausea	2/10	1	1	0	0/10	0	0	0
Malaise	3/10	2	1	0	0/10	0	0	0
Myalgia	0/10	0	0	0	2/10	2	0	0
Arthralgia	1/10	1	0	0	0/10	0	0	0
**20 μg**	**Total**	**Mild**	**Moderate**	**Severe**	**Total**	**Mild**	**Moderate**	**Severe**
Fever	0/10	0	0	0	0/9	0	0	0
Headache	5/10	5	0	0	0/9	0	0	0
Nausea	0/10	0	0	0	1/9	1	0	0
Malaise	0/10	0	0	0	0/9	0	0	0
Myalgia	0/10	0	0	0	0/9	0	0	0
Arthralgia	0/10	0	0	0	0/9	0	0	0
**80 μg**	**Total**	**Mild**	**Moderate**	**Severe**	**Total**	**Mild**	**Moderate**	**Severe**
Fever	0/10	0	0	0	0/9	0	0	0
Headache	1/10	1	0	0	1/9	0	1	0
Nausea	0/10	0	0	0	0/9	0	0	0
Malaise	0/10	0	0	0	0/9	0	0	0
Myalgia	0/10	0	0	0	0/9	0	0	0
Arthralgia	0/10	0	0	0	0/9	0	0	0

Table 4 Anti-AMA1 Specific Antibody Units in Humans Immunized with AMA1-C1/Alhydrogel®, as Determined by ELISA, 14 Days Following Administration of the Second Immunization

Group	Study Participant	Antibody Units	
		Plate Antigen AMA1 3D7	Plate Antigen AMA1 FVO
5 μg	# 1	NR	NR
	# 2	NR	32
	# 3	NR	NR
	# 4	NR	NR
	# 5	211	89
	# 6	NR	NR
	# 7	NR	NR
	# 8	NR	NR
	# 9	68	NR
	# 10	NR	NR
20 μg	# 11	-	-
	# 12	NR	36
	# 13	NR	NR
	# 14	373	333
	# 15	53	101
	# 16	NR	NR
	# 17	53	NR
	# 18	211	112
	# 19	1,440	1,060
	# 20	NR	NR
80 μg	# 21	77	58
	# 22	NR	NR
	# 23	67	89
	# 24	449	287
	# 25	512	455
	# 26	322	269
	# 27	294	347
	# 28	63	108
	# 29	140	33
	# 30	-	-

NR: no response

1.4.4 Previous Human Experience with AMA1 3D7/Montanide ISA 720

A different form of recombinant AMA1 3D7 - expressed in *E. coli* - has also been tested in one other clinical trial in humans. The results of this trial have not been published; however, the following data were obtained via communication with the investigators. A Phase 1 study was conducted in Australia using the recombinant AMA1 ectodomain expressed in and refolded from *E. coli*. Thirty-five (35) healthy adults received either 5 μg (n=10), 20 μg (n=10), or 80 μg (n=9) of protein emulsified in Montanide ISA 720 (a

water-in-oil adjuvant). Immunizations were administered as IM injections in the thigh; the second dose was given 2-3 months following the first dose, and the third dose was given 6 months following the second.

Of the 29 volunteers who received at least one dose of this vaccine, 26 experienced an adverse event (AE) that was either possibly or probably related to the vaccine . A total of 32 AEs that were possibly or probably related to the vaccine occurred over the duration of the trial: one of the AEs was systemic (mild headache and flushing in a volunteer after receiving the first dose of 5 µg), and the remaining events were local reactions consisting mostly of pain and swelling at the injection site (13 mild, 17 moderate, and 1 severe). The 1 severe reaction occurred after the first dose in a volunteer who received 5 µg AMA1, and consisted of severe pain and swelling in the thigh that lasted for 5 days and required treatment with acetylsalicylic acid. In summary, the investigators of this trial concluded that this version of recombinant AMA1 adjuvanted in Montanide ISA 720 was generally tolerated, although a number of volunteers developed local AEs.

1.4.5 Clinical Experience with Aluminum-Based Adjuvants

Several licensed vaccines contain aluminum-based adjuvants, including the recombinant Hepatitis B vaccine (Recombivax HB®), the tetanus toxoid vaccine (TT), and the diphtheria-tetanus toxoids vaccine (DT) [32, 33]. Tetanus toxoid and Recombivax HB® may be particularly useful comparator vaccines: Recombivax HB® consists of a recombinant protein expressed in *Saccharomyces cerevisiae*, whereas tetanus toxoid consists of detoxified *Clostridium tetani* toxin; both are administered intramuscularly. For these two aluminum hydroxide-adsorbed vaccines, local reactions such as pain, tenderness, and swelling are experienced in between 7.6% and 16.7% of volunteers in studies that included over 1,200 healthy adults. Fever is seen in 3.2% to 9.3%, headache in 4.1%, and other systemic symptoms such as fatigue, malaise, nausea, and diarrhea at lower frequencies. Urticaria has been reported in 0.1% of individuals vaccinated with Recombivax HB®.

1.5 Clinical Development Plan

The first AMA1-C1/Alhydrogel® Phase 1 trial started in July 2003, and is being carried out over 66 weeks in the United States. The study is an open-label, dose-escalating Phase 1 clinical trial in healthy adult volunteers designed to evaluate the safety, reactogenicity, and immunogenicity of the AMA1-C1/Alhydrogel® vaccine in malaria-unexposed individuals. The three doses selected for human clinical testing were based on data generated from preclinical studies. The maximum human dose of 80 µg/0.5 mL injected IM was tested in a rabbit toxicology study, and no clinically significant adverse effects were observed. Therefore, this dose was deemed to be a reasonable maximum human dose.

As no significant safety issues have been identified after administration of the first two doses to all dose cohorts in this initial Phase 1 trial we plan to proceed to a malaria-endemic area for Phase 1 and 2 trials. This protocol describes the next phase of clinical testing of this vaccine formulation: a Phase 1 trial in healthy, malaria-exposed adults in a malaria-endemic region. The adult Phase 1 trial is being repeated, given the possibility

that the safety of this vaccine formulation may be different in a population that has been chronically exposed to the antigens contained in this vaccine. Provided no safety concerns are uncovered, age de-escalation, Phase 2, and eventually Phase 3 clinical trials will be undertaken in malaria-endemic areas.

A Phase 1 trial of AMA1-C1/Alhydrogel® in Malian children is being planned for the spring of 2005, assuming no safety issues are identified in the adult study described in this protocol. The decision to proceed to Phase 1 testing in malaria-exposed children will be taken after the interim analysis of this study has been reviewed by the investigators, the Data Safety and Monitoring Board (DSMB), and the sponsor (see **Section 9.2.1**). The interim analysis will include the safety and immunogenicity data of all volunteers following two immunizations, as well as safety data accumulated during the malaria transmission season of 2004 (i.e., up to December 2004). It is anticipated that the interim report will be available for review in early 2005.

It should be stressed that we do not expect the AMA1-C1/Alhydrogel® vaccine to induce sterile immunity (i.e., protect against infection), since it is based on a *P. falciparum* blood-stage antigen that is expressed by the parasite only after having passed through the liver stage of its life cycle. The goal of a blood-stage vaccine is to reduce the severity of the clinical manifestations of infection with the malaria parasite, and thereby reduce morbidity and mortality. Therefore, the presence of infection as determined by either blood smear or parasite DNA detection, should not be seen as evidence of vaccine failure.

1.5.1 Participation of Children

The vaccine candidate being tested in this protocol has not yet been tested in children or malaria-exposed people. It is felt that insufficient data are available to judge the potential risk to children living in malaria-endemic areas. Once safety is established in malaria-exposed adults in Mali, we hope to age de-escalate to children in Mali.

1.6 Comparator Vaccine

1.6.1 Rationale for use of a comparator vaccine

Having a comparator vaccine is particularly useful in Phase 1 trials conducted in malaria-endemic areas, since background immunity and natural exposure to malaria may otherwise make it difficult to interpret immunogenicity data. This is particularly a concern in a setting with seasonal transmission, such as Mali, when immunogenicity assessments may be performed over periods of changing exposure to the malaria parasite, which may affect natural antibody responses to the antigens of interest. Rising titers of antibody to AMA1 could therefore be due to immunization, to natural exposure, or both. The use of a control group will permit comparison of immune responses between vaccinated and unvaccinated volunteers, and will result in a clearer interpretation of serological results. Although a placebo control group would accomplish the same goal, using a comparator vaccine that is beneficial to the subjects increases the benefit-risk ratio, which is always relatively low in Phase 1 trials.

Furthermore, the use of a comparator vaccine in a blinded study reduces potential

investigator bias in reporting adverse events or attributing their causality. It will also safeguard against the probability that an excess of adverse events will be reported in this trial in comparison to an equivalent trial conducted in the United States. From prior experience conducting other trials at this study site, there will be many reported adverse events that are unrelated to the study interventions: this population has reduced access to medical care and performs constant heavy labour compared to a population living in the developed world. Thus, this population has significantly more medical afflictions than would be expected in a study population in the United States. Having a comparator arm in the study enables a fairer assessment of whether or not the rate of adverse events is "normal" or not, since to a reviewer unfamiliar with the study site, viewing the number of adverse events might otherwise lead to an erroneous – and negative – assessment of the vaccine's safety.

1.6.2 Rationale for use of the Hepatitis B vaccine as a comparator

We have chosen Recombivax HB® (Merck & Co., Inc.) as the comparator vaccine for five principal reasons: it is likely to confer some benefit the volunteers receiving it, it has a proven safety record, it is adsorbed to an aluminum-containing adjuvant, as is the AMA1-C1 vaccine, it has the same physical appearanceas the AMA1-C1 vaccine, and its dosing schedule permits easy incorporation into the study design.

Available data suggest that most adults living in rural Mali have not received a primary series of immunizations for Hepatitis B. In Mali as a whole, routine Hepatitis B vaccination for infants began in 2002 when it was incorporated into the Expanded Program for Immunization (EPI). The EPI program was instituted in Donéguébougou (the study site for the trial described in this protocol) less than 6 years ago. It is unlikely that many adults living in Donéguébougou would have received this vaccine. Although data regarding prevalence of hepatitis B infection in Mali is sparse, hepatitis B surface antigen (HBsAg) carriage rates of 15.5% in pregnant women in Bamako, the capital, were reported in 2001 [41]. Although a proportion of potential study participants may have been exposed to hepatitis B and have developed antibodies to HBsAg, vaccination of immune individuals poses no safety hazards.

The recommended dosing schedule for Recombivax HB® for adults is to give a full dose (1.0 mL) at 0, 1, and 6 months by intramuscular injection. However, several studies have demonstrated that increasing the length of time between the second and third doses actually increases the degree of antibody response: Jilg et al compared dosing schedules of 0, 1, and 6 months vs. 0, 1, and 12 months, and found that geometric mean titers of anti-surface antigen antibody were 5,846 IU/l vs. 19,912 IU/l, respectively, after the third injection [37-39]. Thus, although the vaccine was licensed with a schedule of 0, 1, and 6 months, ample evidence suggests that a schedule of 0, 1, and 12 months results in superior antibody responses. Study participants randomized to one of the AMA1-C1/Alhydrogel® arms of the study will be offered Recombivax HB® free-of-charge after the conclusion of the study.

2.0 OBJECTIVES

2.1 Primary Objective

1. To determine the frequency of vaccine-related AEs, graded by severity, for each dose.

2.2 Secondary Objectives

1. To determine the dose of AMA1-C1 that generates the highest antibody concentration at Day 42, as judged by enzyme-linked immunosorbent assay (ELISA).
2. To assess and compare the duration of antibody response to AMA1 FVO and AMA1 3D7.
3. To measure the inhibition of parasite growth as measured by the in vitro GIA to FVO and 3D7.
4. To determine the relationship between anti-AMA1 antibody concentration, as judged by ELISA, and degree of in vitro growth inhibition of *P. falciparum*.
5. To determine the relative specificity of the antibodies to a range of AMA1 serotypes in addition to FVO and 3D7, as judged by ELISA, and growth inhibition on a select panel of parasites with typed AMA1.
6. To perform exploratory studies of B cell populations both before and after immunization.

3.0 STUDY SITE

The study will be conducted in Donéguébougou, a village located 30 km northwest of Bamako, the capital of Mali. It is situated in the sudanian area of Mali. The climate is hot, with daily temperatures ranging from 19 to 40°C. The annual rainfall was 1143.8 mm over 94 days of rain in 1999 and 792.5 mm over 69 days in 2000. Precipitation occurs mainly during the rainy season from June to October/November, which corresponds to the period of intense malaria transmission. However, a small amount of precipitation is also observed in March and April during the early hot dry season, coinciding with a slight increase in the rate in malaria transmission. The Koba River near the village heavily contributes to the persistence of anopheline mosquito breeding sites.

Approximately 1,300 inhabitants live in Donéguébougou, according to the 2003 census. The population is young, with 21.5% aged between 0 and 5 years (see **Table 5**), and 29.5% aged between 18 and 45 years. Yearly census information including family/group identification and a map of the location of compounds and active mosquito breeding sites is available (see **Appendix B** for a study area map). Since 1995, the Malaria Research and Training Center (MRTC) of the University of Bamako has operated a small medical clinic in the village and an adjacent small laboratory, with financial support from the NIH and a US Embassy Self-Help Project. In addition, the MRTC helped set up a primary school that is attended by children from Donéguébougou and neighboring villages. The main economic activity of the population is agriculture and general income levels are very low. Extended families generally live together in separate compounds within the village.

Table 5 The 2003 Donéguébougou population distribution, by age group.

Age range	Population	Proportion (%)	Cumulative Proportion (%)
0-5 years	277	21.47	21.47
6-17 years	488	37.83	59.30
18-45 years	381	29.53	88.84
46-90 years	144	11.16	100.00
**Total**	**1290**	**100.00**	

Donéguébougou has been chosen as a site for testing malaria vaccines based on examination of malariometric indicators collected over several transmission seasons (including infection rates, disease prevalence and incidence, entomologic inoculation rates [EIRs], and demographic data) and a close relationship between the MRTC and the community.

In ongoing studies performed by MRTC personnel in Donéguébougou, malaria clinical episodes have occurred more frequently in children aged less than 5 years old, with an attack rate of 95% over the 1999-2000 transmission season, and have decreased significantly with age (87% in 6-10 year-olds, 78% in 11-15 year-olds, and only 52% in 16-20 year-olds [p-value for trend <0.01]). This trend was observed in both 1999 and 2000, and the overall incidence of malaria has remained stable over 3 successive years of observation. The mean number of malaria episodes per child was 1.92 in 2000; however, some children experienced up to 5 clinical episodes of malaria in this transmission season. The parasite prevalence rate in children between the ages of 2 and 9 was 71.8% in July 1999. *P. falciparum* represented 93 to 97% of infecting *Plasmodium* species, while *P. malariae* represented less than 8%, and *P. ovale* less than 1% of all malaria infections.

As in the rest of Mali, malaria transmission in Donéguébougou is seasonal, with significant variability in the EIR over the course of a season. For example, the EIR was nil in June 1999 but reached 7.08 infective bites per person per month in November 1999, as determined by the spray-catch technique. In 2000 the peak EIR – 7.78 – was observed in October. The cumulative EIR over 6 months of surveys from June to December 1999 and 2000 was 21.15 and 19.23 infective bites per person, respectively, by the spray catch technique, indicating a stable EIR from year to year. Transmission is mainly effected by *Anopheles gambiae s.l.* which constitutes 98.2% of the mosquito vector population. *A. funestus* represents 1.8% of the vector population and has a more important role in transmission during the early dry hot season when the local ecosystem favors the persistence of breeding sites suitable to this species. Typically, the transmission season starts in June or July and continues through November/December, with a second, extremely low, transmission season in March/April that is dependent upon *A. funestus*.

An MRTC clinical research team has been on-site in Donéguébougou since 1998. The team is constituted of medical doctors, pharmacists, pre-doctoral medical students and local native guides. This team maintains the operation of the local medical clinic that provides health care to all of the Donéguébougou populace, in addition to the populations from surrounding villages. Through the conduct of several cross-sectional and longitudinal studies, the team has achieved a high level of competency and expertise. Additionally, team members are involved in most social events occurring in Donéguébougou ensuring frequent interaction with community members. This has resulted in a strong social connection between the research team and the community and has helped to enhance compliance to follow-up schedules. In addition, MRTC clinical activities are conducted in conformity with district health policies. The district health officer participates in the evaluation of health care activities, and Donéguébougou health records are integrated into the district health statistics.

As part of the preparation of this site for vaccine trials, the NIAID has sponsored several longitudinal studies in Donéguébougou that were conducted by investigators from the MRTC/DEAP/FMPOS of the Université de Bamako, from 1998 to the present. In 1998, a cohort of children and young adults between 7 months to 20 years of age were followed from July to December to measure the incidence of asymptomatic and symptomatic malaria infection. Subsequently, in 1999 and 2000, approximately 200 volunteers aged 3 months to 21 years were followed to determine the incidence of malaria infection and disease. Starting in July 2001, a subset of this original cohort, consisting of volunteers aged 6 months to 6 years, continued to be followed; half received chloroquine-proguanil chemoprophylaxis for the duration of the malaria transmission season to determine its effect on malaria disease and anemia. In all of these studies, study participants were followed actively on a weekly basis and passively through the on-site availability of study physicians to evaluate any medical complaint at any time during the study period. Loss to follow-up in these cohorts was usually less than 5% per year.

Finally, in July 2002, a study consisting of repeated cross-sectional surveys was started; a total of about 500 children and adults are presently enrolled in this study (NIAID protocol number 02-I-N248). Study participants are seen four times through the year, at which points demographic and clinical information, as well as blood, are collected. The aims of this study are to establish reference intervals for certain hematologic and biochemistry parameters, to determine natural humoral immune responses to several *P. falciparum* antigens, and to determine *P. falciparum* gene polymophisms.

4.0 STUDY DESIGN

4.1 Overall Design

The study is a randomized, controlled, Phase 1 dose-escalating clinical trial in healthy, malaria-exposed adult volunteers designed to evaluate the safety, reactogenicity, and immunogenicity of the AMA1-C1 malaria vaccine formulated on Alhydrogel®, as compared to Recombivax HB® Hepatitis B vaccine. The study will be divided into two parts: the first part will be double-blinded and study the safety and immunogenicity of AMA1-C1/Alhydrogel®, following vaccinees through a single malaria transmission

season. At the completion of this part of the study, the randomization code will be revealed to the investigators and an interim report detailing the safety and immunogenicity to Day 180 will be prepared (see **Sections 8.6** and **9.2.1**). This report will form the basis for decisions to proceed to further studies with this formulation, such as Phase 1 studies in children. The second part of the study will be single-blinded (i.e., participants will not know to which group they have been randomized). Safety parameters will continue to be monitored. The primary immunological endpoint will be the measurement of the longevity of the immune response and the ability of this response to be boosted.

After obtaining the consent and cooperation of the village leaders and local officials, volunteers will be invited to participate in the study. After providing written informed consent, volunteers will undergo eligibility screening, including medical history, physical examination, hematology testing, liver and renal function testing, Hepatitis B and C screening, and urinalysis; urine pregnancy testing will be performed on all female volunteers. All clinically significant abnormalities will be reviewed with volunteers and referral for follow-up care will be provided. After screening, those volunteers determined to be eligible, based on the inclusion and exclusion criteria described in **Section 5.0** in this protocol, will be invited to participate in the study.

Dose cohorts will be enrolled and vaccinated in a consecutive fashion as described in **Section 4.4**; that is, the first dose cohort will be assembled first, followed by the second, and then the third. Fifty-four volunteers will be progressively enrolled into one of three dose cohorts. The 18 volunteers in each cohort will be subsequently randomized in a 2:1 ratio to receive either the AMA1-C1/Alhydrogel® vaccine (n=12) or Recombivax HB® (n=6). Volunteers randomized to receive the AMA1-C1 vaccine will receive either the 5 µg, 20 µg, or 80 µg dose of AMA1-C1 as outlined in **Table 6**.

As with other aluminum hydroxide-adsorbed vaccines, hypersensitivity reactions would be expected to occur within the first 24 hours after receipt of either of the two vaccines, and other severe local or systemic reactions within 72 hours of vaccination. Volunteers will therefore be observed for immediate reactions following each vaccination for 30 minutes, and will return to the study clinic on Days 1, 2, 3, 7, and 14 following each vaccination for clinical assessment. See **Table 6** for a tabular description of the vaccination schedule for all 3 dose cohorts, as well as **Section 7.6** and **Appendix D** for a detailed description of the scheduled clinical and laboratory evaluations.

Prior to dose escalation, safety data up to and including Day 7 post-vaccination will be available from the lower dose cohort for review by the Medical Monitor and/or Data Safety and Monitoring Board (DSMB). The trial will not proceed to the next dose cohort if, in the clinical judgment of the DSMB and/or Medical Monitor, the next higher dose would pose an unacceptable safety risk to the volunteers (see Section **8.5**).

Table 6 Dose Escalation Schedule

**Time**[1] (Calendar Day)	**Cohort 1** (n = 18)<br><br>5 µg AMA1-C1<br>800 µg Alhydrogel®<br>424 µg Al[2]<br>(n=12)<br>**OR**<br>Recombivax HB® (n=6)	**Cohort 2** (n = 18)<br><br>20 µg AMA1-C1<br>800 µg Alhydrogel®<br>424 µg Al[2]<br>(n=12)<br>**OR**<br>Recombivax HB® (n=6)	**Cohort 3** (n = 18)<br><br>80 µg AMA1-C1<br>800 µg Alhydrogel®<br>424 µg Al[2]<br>(n=12)<br>**OR**<br>Recombivax HB® (n=6)
0	Vaccination 1 (Day 0)		
20		Vaccination 1 (Day 0)	
28	Vaccination 2 (Day 28)		
40			Vaccination 1 (Day 0)
48		Vaccination 2 (Day 28)	
68			Vaccination 2 (Day 28)
360	Vaccination 3 (Day 360)		
380		Vaccination 3 (Day 360)	
400			Vaccination 3 (Day 360)

[1]Approximate (see Section 7.6 for more detail)
[2]Aluminum content per dose

4.2 Sample Size and Estimated Duration of Study

A total of fifty-four volunteers will be enrolled (see **Section 10.2** for a justification of the sample size). Thirty-six will receive the AMA1-C1/Alhydrogel® vaccine (one of three dose concentrations), and 18 will receive Recombivax HB®. The trial is expected to last for a total of 83 weeks. Each volunteer will be followed for 78 weeks from the time of the first injection.

4.3 Timing of Third Vaccination (Day 360)

The third dose of vaccine will be administered on Study Day 360 for each of the three dose cohorts. The timing of this booster dose was chosen to roughly correspond to the start of the 2005 malaria transmission season. The first two doses of vaccine will be administered just prior to the 2004 malaria transmission season, which usually starts in July. Between the second and third immunization in this study, the cumulative safety data from the Phase 1 trial of the AMA1-C1/Alhydrogel® vaccine taking place in the United States will be reviewed, as all volunteers in that study will have received all three immunizations by that point. The third dose will be administered in Mali only if there are no safety concerns raised from the trial in the United States.

Vaccinations in the Phase 1 trial of AMA1-C1/Alhydrogel® that is presently being conducted in the United States are being given on Days 0, 28, and 180. Day 180 was chosen for the third vaccination in order to demonstrate boosting of the immune response; whether this is demonstrated after six months or twelve months isn't considered

to be of great significance. Therefore, in the US study, a six-month boost was chosen for practical reasons (to reduce the duration of the trial, retain study participants, etc.). However, in Mali, the timing of the third dose becomes more relevant. If boosting is required for the AMA1-C1/Alhydrogel® vaccine, it would be logical to time the boost to occur immediately prior to the next seasonal exposure to the malaria parasite. Volunteers will subsequently be followed throughout the 2005 malaria transmission season (until December 2005), to exhaustively monitor for safety concerns that may arise due to chronic exposure to the parasite after vaccination.

4.4 Group Allocation

The three dose cohorts will be enrolled consecutively. Within each cohort of 18 volunteers, 12 will be randomly assigned to receive AMA1-C1/Alhydrogel® vaccine and 6 to receive Recombivax HB® (see **Section 7.3**). Within the first, second and third dose cohort ("Cohort 1", "Cohort 2", and "Cohort 3"), those randomized to receive the AMA1-C1/Alhydrogel® vaccine will receive that which contains 5 μg, 20 μg, and 80 μg of AMA1-C1, respectively.

Once screening has started and the first 20 participants have been deemed eligible, they will be assigned to dose Cohort 1, and a Day 0 visit will be scheduled. On the day of vaccination, the first 18 who arrive at the study clinic will be randomly assigned in a 2:1 fashion to receive either the AMA1-C1/Alhydrogel® vaccine (5 μg) or Recombivax HB®; the remaining 2 will be kept as alternates if some of the first 18 cannot be vaccinated on the day of first vaccination (i.e., due to pregnancy, withdrawal of consent, etc.). If the alternates are not vaccinated, they will be invited to participate as members of the next dose cohort.

Following assembly of the first dose cohort, the next 20 eligible volunteers to be screened will be assigned to dose Cohort 2 and their Day 0 visit scheduled using the same procedure as described for the first dose cohort. Similarly, the next 20 eligible volunteers screened following assembly of Cohort 2 will be assigned to Cohort 3 and their Day 0 visit scheduled as per the previous two cohorts.

4.5 Blinding

For the first 6 months of the study, investigators and participants will be blinded as to an individual study participant's allocation to either AMA1-C1/Alhydrogel® vs. Recombivax HB® vaccine; only the study drug manager/pharmacist and their assistant will be aware of this allocation. The study drug manager/pharmacist will refer to the unique randomization code assigned to that participant to determine the assigned vaccine for each participant. Vaccine will be prepared by the study pharmacist in a separate room and the vaccine-filled syringes will be passed to vaccinators through a small window separating the vaccine preparation room and the vaccination rooms. Since the AMA1-C1/Alhydrogel® and Recombivax HB® vaccines are of different volumes (0.5 mL and 1.0 mL, respectively), the contents of the syringes will be disguised using opaque tape. As a further precaution, the individuals administering the vaccine will not be involved in assessments of reactogenicity or adverse events.

Due to the staggered, dose-escalation design of the trial, it will not be possible to blind to the dose of AMA1-C1 that an individual may have received. That is, those participants assigned to Cohort 1 may receive either the 5 μg dose of AMA1-C1/Alhydrogel® or the Recombivax HB® vaccine, whereas Cohort 2 will receive either the 20 μg dose of AMA1-C1/Alhydrogel® or Recombivax HB®, and Cohort 3 will receive either the 80 μg dose of AMA1-C1/Alhydrogel® or Recombivax HB®. Since Cohort 1 will receive their vaccinations before Cohort 2 (see **Table 6**), it will not be possible to blind to the dose of AMA1-C1 that may have been given, only to the allocation between AMA1-C1/Alhydrogel® vs. Recombivax HB®.

The investigators will be unblinded to participants' vaccine allocation status after all have had their study Day 180 visit (see **Section 8.6).** After this point, the study will be single-blinded (i.e., study participants will remain blinded to what vaccine they have received). The principal justification for unblinding the investigators before the third immunization has been administered is that the safety and immunogenicity data acquired from the first two immunizations will be crucial in guiding our clinical development plan for this product, and we feel that, to make an informed decision as to whether or not to proceed to pediatric trials, we must first assess the unblinded data. Furthermore, the primary purpose of the third immunization on Day 360 is to determine whether antibody levels can be boosted immediately prior to a subsequent malaria transmission season. Thus, maintaining the study blind for this immunization is not as necessary as for the first part of the study, when obtaining completely unbiased assessments of vaccine safety is the primary goal.

5.0 SELECTION AND ENROLLMENT OF VOLUNTEERS

5.1 Inclusion Criteria

1. Males or females between 18 and 45 years, inclusive.
2. Known residents of the village of Donéguébougou, Mali.
3. Good general health as determined by means of the screening procedure.
4. Available for the duration of the trial (78 weeks).
5. Willingness to participate in the study as evidenced by signing the informed consent document.

5.2 Exclusion Criteria

1. Pregnancy as determined by a positive urine β-hCG (if female).
2. Participant and her spouse are unwilling to use reliable contraception methods up until one month following the third immunization (if female).
3. Currently lactating and breast-feeding (if female).
4. Evidence of clinically significant neurologic, cardiac, pulmonary, hepatic, rheumatologic, autoimmune, chronic infectious, or renal disease by history, physical examination, and/or laboratory studies including urinalysis.
5. Behavioral, cognitive, or psychiatric disease that in the opinion of the investigator affects the ability of the volunteer to understand and cooperate with the study protocol.

6. Laboratory evidence of liver disease (alanine aminotransferase [ALT] greater than 1.25 times the upper limit of normal of the testing laboratory).
7. Laboratory evidence of renal disease (serum creatinine greater than the upper limit of normal of the testing laboratory, or more than trace protein or blood on urine dipstick testing).
8. Laboratory evidence of hematologic disease (absolute leukocyte count $<3000/mm^3$ or $>13.5 \times 10^3/mm^3$; hemoglobin < 0.9 times the lower limit of normal of the testing laboratory, by sex; absolute lymphocyte count $<1000/mm^3$; or platelet count $<120{,}000/mm^3$).
9. Other condition that in the opinion of the investigator would jeopardize the safety or rights of a volunteer participating in the trial or would render the subject unable to comply with the protocol.
10. Participation in another investigational vaccine or drug trial within 30 days of starting this study, or while this study is ongoing.
11. Volunteer has had medical, occupational, or family problems as a result of alcohol or illicit drug use during the past 12 months.
12. History of a severe allergic reaction or anaphylaxis.
13. Severe asthma (emergency room visit or hospitalization within the last 6 months).
14. Positive ELISA for HCV.
15. Positive HBsAg by ELISA.
16. Known immunodeficiency syndrome.
17. Use of corticosteroids (excluding topical or nasal) or immunosuppressive drugs within 30 days of starting this study or while the study is ongoing.
18. Receipt of a live vaccine within past 4 weeks or a killed vaccine within past 2 weeks prior to entry into the study.
19. History of a surgical splenectomy.
20. Receipt of blood products within the past 6 months.
21. Previous receipt of an investigational malaria vaccine.
22. History of a known allergy to nickel.
23. Previous receipt of a primary series of any Hepatitis B vaccine.
24. History of known allergy to yeast.

5.2.1 Rationale for Use of Clinical Assessments of Immunosuppression

We do not plan to test for HIV at the time of screening for two reasons. First, HIV seroprevalence is 1.7% in Mali, one of the lowest rates in sub-Saharan Africa. Although no serosurveys have been done in Donéguébougou itself, this site is in a rural area not easily accessible by road, and almost certainly has a lower prevalence rate than the average for the entire country. After working at this site for the past nine years, we have not encountered anyone with an illness that raised clinical suspicion of an immunosuppressive disease. Therefore, the training of staff and establishment of programs that would be necessary for voluntary counseling and testing for HIV would likely yield few, if any, cases of HIV in this small study. Second, the potential stigma that may result from being diagnosed with HIV is considered too great a risk in this tightly-knit community.

5.3 Treatments That Could Potentially Interfere with Vaccine-Induced Immunity

The following criteria should be checked at each visit. If any become applicable during the study, the participant will be excluded from receiving further doses of the study vaccine and will not be included in the immunogenicity evaluations after the time of exclusion. The participant will, however, be encouraged to remain in the safety evaluation for doses already received.

1. Use of any investigational drug or investigational vaccine other than the study vaccine during the study period.
2. Administration of chronic (defined as more than 14 days) immunosuppressants or other immune-modifying drugs 6 months prior to vaccination. (Topical and nasal steroids are allowed.)
3. Administration of a licensed vaccine during the period starting from Day -14 to Day 42 or from Day 346 to Day 374 (14 days before and after each vaccination).
4. Administration of immunoglobulins and/or any blood products up to 30 days after the last dose of vaccine.

5.4 Contraindications to Vaccination

The following criteria should be checked prior to each immunization and are contraindications to further immunization. However, the participant will be encouraged to remain in the safety evaluation for doses already received.

1. Hypersensitivity reaction following administration of the study vaccine.
2. Pregnancy, as determined by a positive urine β-hCG.
3. Occurrence of severe chronic disease (such as diabetes or tuberculosis), which in the view of the investigators or Medical Monitor could jeopardize the safety of study participant or may complicate interpretation of the safety or immunogenicity data.

5.5 Indications for Deferral of Vaccination

The following adverse events (AEs) constitute grounds for deferral of vaccine administration at that point in time; if any one of these AEs occurs at the time scheduled for vaccination, the participant may be vaccinated at a later date, within the allowable time interval specified in **Section 7.6** of this protocol, or withdrawn at the discretion of the investigator. The participant must be followed until resolution of the event as with any AE. If the participant is withdrawn from the study, he/she will be encouraged to remain in the safety evaluation for the duration of the study.

1. Oral temperature > 37.5°C or other evidence of clinical malaria at the time of vaccination will warrant deferral of immunization until fever and symptoms resolve.
2. Any other acute condition that in the opinion of the investigator poses a threat to the individual if immunized or that may complicate interpretation of the safety of the vaccine following immunization.

Such individual(s) will be followed in the clinic until the symptoms resolve or the window for immunization expires. No further vaccination will be performed if the participant does not recover (oral temperature ≤ 37.5°C and/or lack of symptoms) within the originally scheduled vaccination time interval. The participant, however, will be followed for safety and immunogenicity evaluations. If the individual meets any of the above criteria for deferral on the day of first immunization, as an alternative to deferral of vaccination, the investigator may instead elect to exclude the participant from further participation in the study. Eligible alternates will then be vaccinated instead.

5.6 Subject Withdrawal Criteria

A volunteer will not be considered to have completed the trial if any of the following reasons apply. However, any volunteer who has received at least one dose of vaccine will be encouraged to remain in the safety evaluation for the duration of the study. Should a female volunteer become pregnant during the course of the study, she will be followed for the duration of the pregnancy.

1. *Research terminated by sponsor or investigator* – applies to the situation where the entire study is terminated by the sponsor, or investigator for any reason.
2. *Withdrawal of consent* – applies to a subject who withdraws consent to participate in the study for any reason.
3. *Noncompliant with protocol* – applies to a volunteer who does not comply with protocol-specific visits or evaluations, on a consistent basis, such that adequate follow-up is not possible and the volunteer's safety would be compromised by continuing in the trial. Additionally, this applies to a volunteer who is lost to follow-up and cannot be located.
4. *Other* – is a category used when previous categories do not apply, and requires an explanation.

6.0 VACCINE PREPARATION

6.1 Supplies

The AMA1-C1/Alhydrogel® research products for this protocol will be supplied to the study-site pharmacist by the Pharmaceutical Development Section, Pharmacy Department, Clinical Center, National Institutes of Health, where the AMA1-C1/Alhydrogel® vaccine was formulated and vialed. Both the AMA1-C1/Alhydrogel® and Recombivax HB® vaccines will be transported to Mali at 0.5°C to 9°C; temperature recording devices will accompany the vaccines at all times to ensure storage temperature limits have not been violated. Vaccine will be stored at the FMPOS in Bamako in a refrigerator at 0.5°C to 9°C and will not be frozen; refrigerator temperature will be monitored continuously. One to two days prior to vaccination, adequate supplies of vaccine will be transported to the study site in temperature-monitored coolers; at the study site, they will be stored in a temperature-monitored refrigerator (powered by a generator with double backup). One day after all participants in a given cohort have been vaccinated, unused vials of vaccine will be transported back to the FMPOS for storage until the next vaccination day. Single-dose vials will be stored in the upright position.

AMA1-C1/Alhydrogel® malaria vaccine is supplied as a slightly turbid suspension in single-dose vials. Each 2.0 mL vial contains 0.7 mL, of which 0.5 mL is the intended volume to be injected. 0.5 mL of vaccine contains the equivalent of 424 µg of aluminum as Alhydrogel® (800 µg of aluminum hydroxide gel per dose) onto which either 5 µg, 20 µg, or 80 µg of recombinant AMA1-C1 has been bound. The product conforms to established requirements for sterility, safety, and identity.

Recombivax HB® (Merck & Company, Inc.) is a non-infectious subunit viral vaccine derived from hepatitis B surface antigen (HBsAg) produced in yeast cells. The antigen is harvested and purified from fermentation cultures of a recombinant strain of the yeast *Saccharomyces cerevisiae* containing the gene for the *adw* subtype of HBsAg. The purified protein is treated in phosphate buffer with formaldehyde and then coprecipitated with alum (potassium aluminum sulfate) to form bulk vaccine adjuvanted with amorphous aluminum hydroxyphosphate sulfate. Recombivax HB® is a slightly turbid sterile suspension for intramuscular injection. Each 1.0 mL dose contains 10 µg of hepatitis B surface antigen and approximately 500 µg of aluminum (provided as amorphous aluminum hydroxyphosphate sulfate). The product conforms to established requirements for sterility, safety, and identity.

6.2 Vaccine Storage

Both AMA1-C1/Alhydrogel® and Recombivax HB® should be maintained at 0.5°C to 9°C until just prior to administration. Vaccine should NOT be frozen at any time.

6.3 Vaccine Accountability

Study-site pharmacists are responsible for maintaining an accurate inventory and accountability record of vaccine supplies for this study. Partially used vials may not be administered to other volunteers.

6.4 Disposition of Used/Unused Supplies

After administration of a vaccine dose, the single-dose vial will be stored in the study pharmacy at the study site, and vials will be accounted for and stored until monitoring by the study sponsor. The vials may then be disposed of according to site protocol. At the conclusion of the study, all unused AMA1-C1/Alhydrogel® vaccine supplies will be destroyed on site, or returned to the Pharmaceutical Development Section, Pharmacy Department, Clinical Center, National Institutes of Health, who will return them to the MVDB or destroy them, as requested by the Sponsor. All other vials will be retained until requested.

7.0 STUDY PROCEDURES

The following sections provide a detailed listing of the procedures and studies to be performed in this protocol at designated time points. The total volume of blood (approximately 495 mL) to be drawn over the 18-month duration of the trial is less than the volume collected when donating two units of blood and should not compromise the health of trial participants.

7.1 Community Consent

The previous studies conducted by the MRTC in Donéguébougou (as described in **Section 3.0**) have permitted extensive contact with the village population that has led to the development of mutual trust and the establishment of an ongoing informed consent process attempting to address issues related to interventional studies in resource-limited settings. Many discussions with village leaders, heads of families and villagers through group meetings, and more limited group interviews have reviewed the need to obtain a written informed consent from study participants. The community has now become familiar with the informed consent process, including written, signed consent forms.

The community informed consent process goes through the following steps:

i. Explanation and clarification to village leaders, including the village chief and elders.
ii. Allow time for village leaders to communicate with community members and relay any additional questions or concerns.
iii. Take time to explain protocols to heads of families.

Prior to administering individual informed consent, the study team conducts careful word-for-word review of the study consent form that will be translated orally into local languages and dialects in the event that a potential study participant does not read or speak French (which will likely be a majority of the potential participants). Verification that the oral translations are accurate and that the potential participants understand the contents of the informed consent form will be done by the independent witness as described in **Section 7.2**.

7.2 Individual Recruitment and Informed Consent

Volunteers aged 18-45 years, inclusive, will be invited through a general announcement in the village to come to the study clinic for screening. During this initial screening visit, the volunteer will read the consent form or have it explained to him/her in cases of illiteracy. Volunteers will be encouraged to ask questions, and then take a multiple-choice questionnaire to evaluate consent comprehension (**Appendix C**); this will be administered orally in the case of potential volunteers who cannot read. The volunteer must answer all questions correctly prior to being eligible for enrollment. Study staff will use incorrect answers from the questionnaire to identify those areas of the informed consent form that need further review with the volunteer. This will help ensure the volunteer has sufficient understanding before the consent form is signed. The volunteer may either sign the consent form immediately or later after further consideration. Volunteers unable to read will place an imprint of their finger in the place of a signature; in addition, an independent witness will sign the consent form to attest that the volunteer fully comprehended the contents.

The following procedures will be performed upon initial screening (note that all procedures might not be performed on the same day):

1. Explain the study and Informed Consent to the volunteer.

2. Ensure the subject has signed the Informed Consent and receives a signed copy of the Informed Consent and has passed the informed consent comprehension exam.
3. Elicit a complete medical history, including menstrual and contraceptive history and/or history of surgical sterility for female subjects.
4. Administer a complete physical examination.
5. Obtain approximately 15 mL of blood for hematology, biochemistry, and serologic tests for viral hepatitis in all volunteers.
6. Obtain urine for urine dipstick testing, as well as urine β-hCG testing in females.
7. Counsel females to avoid becoming pregnant during the study.

Any clinically relevant finding that is discovered upon screening will be treated appropriately according to the standard of care in Mali, as follows. Initial management will be performed at the study clinic, free of charge. Should referral for more extensive investigation or treatment be required, the study will arrange and pay for transportation to one of the National Hospitals and initial consultation. Initial care – according to the standard of care in Mali – will be covered by the study; however, in the event that a chronic illness is discovered during the course of screening, long-term treatment and care will not be reimbursed by the study.

7.3 Randomization Process

The eligible volunteers assigned to each of the three cohorts will be asked to come to the study clinic on their scheduled day of enrollment into the study. After undergoing a clinical interview and exam to ensure that they remain eligible for participation in the study, they have had blood collected for the studies outlined in **Section 7.6**, and females have had a negative urine pregnancy test performed, volunteers will be vaccinated as described in **Section 7.5**.

Study participants will be assigned a unique study number and will be given a photo identification card to aid in their identification; a copy of the photo identification card will be place in the participant's study file.

Randomization to either the AMA1-C1/Alhydrogel® vaccine or Recombivax HB® will be done through use of a randomization envelopes. The randomization envelopes will be prepared in advance of the start of the study and will contain sequential codes linking a study number to a vaccine assignment (AMA1-C1 or Recombivax HB®). The study numbers will be assigned in the order in which the participants are enrolled in the study, so that among the first 18 study numbers, 12 will be assigned to AMA1-C1 whereas 6 will be assigned to Recombivax HB®. Assignment of the study numbers will be done on the day of first vaccination, in the order that the study participants present for immunization.

Access to the randomization list will be exclusively limited to the study drug manager(s)/pharmacist(s). On days of vaccination, only the study drug manager(s)/pharmacist(s) will have access to the vaccine preparation room, which will be separated from the vaccination rooms by windows with sliding doors through which prepared vaccine will be passed. Between vaccination days, the randomization list will

be stored in a locked cabinet in the vaccine preparation room. The study drug manager(s)/pharmacist(s) will be unblinded, but will not be involved in study participants' further evaluation. The Medical Monitor will also keep one set of the randomization code in a sealed envelope in the event that emergency unblinding is required (see **Section 8.6**), and one set will be sent to the DSMB.

7.4 Enrollment

Volunteers will not be considered enrolled in the study until they have received their first dose of vaccine.

7.5 Immunization Procedure

Volunteers will receive three immunizations, on Days 0, 28, and 360. Both AMA1-C1/Alhydrogel® vaccine and Recombivax HB® will be kept refrigerated at 0.5°C to 9°C until just before use, whereupon they will be warmed to room temperature. 0.5 mL (for the AMA1-C1/Alhydrogel® vaccine) or 1.0 mL (for the Recombivax HB® vaccine) will be delivered by IM injection in the deltoid muscle with a 22-gauge needle of appropriate length after preparation of the site with alcohol. Successive vaccinations will be given in alternating arms.

7.6 Clinical Monitoring and Evaluation

See **Appendix D** for a tabular representation of study procedures.

<u>Study Day 0 (Day of First Vaccination)</u>

1. Verify that Informed Consent was obtained.
2. Verify that all applicable eligibility criteria have been met.
3. Perform abbreviated history and physical exam, focusing on any acute complaints.
4. Obtain approximately 55 mL of blood for hematology, biochemistry, anti-AMA1 antibody ELISA, B cell analysis, filter paper collection, and GIA.
5. For females, obtain a urine sample for β-hCG testing. Ensure that test is negative before proceeding; a positive test will exclude the volunteer from the trial.
6. Record vital signs (blood pressure, temperature, heart rate, and respiratory rate).
7. Administer the vaccine.
8. Observe for 30 minutes after vaccination to evaluate for immediate adverse reactions. During the 30-minute post-immunization wait period, study staff will discuss signs and symptoms of potential AEs.

Study Day 1

1. Perform basic history and physical exam (including injection site), emphasizing examination of any acute complaints.
2. Record vital signs.

Study Day 2

1. Perform basic history and physical exam (including injection site), emphasizing examination of any acute complaints.
2. Record vital signs.

Study Day 3

1. Perform basic history and physical exam (including injection site), emphasizing examination of any acute complaints.
2. Record vital signs.
3. Obtain approximately 20 mL of blood for hematology and biochemistry tests, and B cell analysis.

Study Day 7 +/- 1

1. Perform basic history and physical exam (including injection site), emphasizing examination of any acute complaints.
2. Record vital signs.
3. Obtain 15 mL of blood for hematology and B cell analysis.

Study Day 14 +/- 2

1. Perform basic history and physical exam (including injection site), emphasizing examination of any acute complaints.
2. Record vital signs.
3. Obtain approximately 25 mL of blood for hematology, biochemistry, filter paper collection, anti-AMA1 antibody ELISA, and B cell analysis.

Study Day 28 +/- 7 (Day of Second Vaccination)

1. Perform basic history and physical exam (including injection site), emphasizing examination of any acute complaints.
2. Obtain approximately 25 mL of blood for hematology, biochemistry, filter paper collection, anti-AMA1 antibody ELISA, and B cell analysis.
3. For females, obtain a urine sample for β-hCG testing. Ensure that test is negative before proceeding; a positive test will exclude the volunteer from the trial.
4. Record vital signs (blood pressure, temperature, heart rate, and respiratory rate).
5. Administer the vaccine.
6. Observe for 30 minutes after vaccination to evaluate for immediate adverse reactions. During the 30-minute post-immunization wait period, study staff will discuss signs and symptoms of potential AEs.

Study Day 29 (1 day after Second Vaccination)

1. Perform basic history and physical exam (including injection site), emphasizing examination of any acute complaints.
2. Record vital signs.

Study Day 30 (2 days after Second Vaccination)
1. Perform basic history and physical exam (including injection site), emphasizing examination of any acute complaints.
2. Record vital signs.

Study Day 31 (3 days after Second Vaccination)
1. Perform basic history and physical exam (including injection site), emphasizing examination of any acute complaints.
2. Record vital signs.
3. Obtain approximately 20 mL of blood for hematology and biochemistry tests, and B cell analysis.

Study Day 35 +/- 1 (7 days after Second Vaccination)
1. Perform basic history and physical exam (including injection site), emphasizing examination of any acute complaints.
2. Obtain 15 mL of blood for hematology and B cell analysis.
3. Record vital signs.

Study Day 42 +/- 2 (14 days after Second Vaccination)
1. Perform basic history and physical exam (including injection site), emphasizing examination of any acute complaints.
2. Record vital signs.
3. Obtain approximately 40 mL of blood for hematology, biochemistry, anti-AMA1 antibody ELISA, B cell analysis, filter paper collection, and GIA.

Study Day 60 +/-7 (1 month after Second Vaccination)
1. Perform basic history and physical exam (including injection site), emphasizing examination of any acute complaints.
2. Obtain approximately 5 mL of blood for hematology analysis and filter paper collection.

Study Day 90 +/- 10 (2 months after Second Vaccination)
1. Perform basic history and physical exam, emphasizing examination of any acute complaints.
2. Obtain approximately 10 mL of blood for hematology analysis, filter paper collection, and anti-AMA1 antibody ELISA.

Study Day 120 +/-10 (3 months after Second Vaccination)

1. Perform basic history and physical exam, emphasizing examination of any acute complaints.
2. Obtain approximately 5 mL of blood for hematology analysis and filter paper collection.

Study Day 150 +/- 14 (4 months after Second Vaccination)
1. Perform basic history and physical exam, emphasizing examination of any complaints.
2. Record vital signs.
3. Obtain approximately 10 mL of blood for hematology, filter paper collection, and anti-AMA1 antibody ELISA.

Study Day 180 +/- 14 (5 months after Second Vaccination)
1. Perform basic history and physical exam, emphasizing examination of any complaints.
2. Obtain approximately 35 mL of blood for hematology, anti-AMA1 antibody ELISA, B cell analysis, filter paper collection, and GIA.

Study Day 270 +/- 30
1. Perform basic history and physical exam, emphasizing examination of any complaints.
2. Record vital signs.
3. Obtain approximately 15 mL of blood for hematology, biochemistry, filter paper collection, and anti-AMA1 antibody ELISA.

Study Day 360 +/- 30 (Day of Third Vaccination)
1. Perform basic history and physical exam (including injection site), emphasizing examination of any acute complaints.
2. Obtain approximately 40 mL of blood for hematology, biochemistry, anti-AMA1 antibody ELISA, filter paper collection, GIA, and B cell analysis.
3. For females, obtain a urine sample for β-hCG testing. Ensure that test is negative before proceeding; a positive test will exclude the volunteer from the trial.
4. Record vital signs (blood pressure, temperature, heart rate, and respiratory rate).
5. Administer the vaccine.
6. Observe for 30 minutes after vaccination to evaluate for immediate adverse reactions. During the 30-minute post-immunization wait period, study staff will discuss signs and symptoms of potential AEs.

Study Day 361 (1 day after Third Vaccination)
1. Perform basic history and physical exam (including injection site), emphasizing examination of any acute complaints.
2. Record vital signs.

Study Day 362 (2 days after Third Vaccination)
1. Perform basic history and physical exam (including injection site), emphasizing examination of any acute complaints.

2. Record vital signs.

Study Day 363 (3 days after Third Vaccination)
1. Perform basic history and physical exam (including injection site), emphasizing examination of any acute complaints.
2. Record vital signs.
3. Obtain approximately 20 mL of blood for hematology and biochemistry tests, and B cell analysis.

Study Day 367 (7 days +/- 1 after Third Vaccination)
1. Perform basic history and physical exam (including injection site), emphasizing examination of any acute complaints.
2. Obtain 15 mL of blood for hematology and B cell analysis.
3. Record vital signs.

Study Day 374 (14 days +/- 2 after Third Vaccination)
1. Perform basic history and physical exam (including injection site), emphasizing examination of any acute complaints.
2. Record vital signs.
3. Obtain approximately 40 mL of blood for hematology, biochemistry, anti-AMA1 antibody ELISA, B cell analysis, filter paper collection, and GIA.

Study Day 390 (1 month +/-10 days after Third Vaccination)
1. Perform basic history and physical exam, emphasizing examination of any acute complaints.
2. Obtain approximately 5 mL of blood for hematology analysis and filter paper collection.

Study Day 420 (2 months +/-14 days after Third Vaccination)
1. Perform basic history and physical exam, emphasizing examination of any acute complaints.
2. Obtain approximately 10 mL of blood for hematology, filter paper, and anti-AMA1 antibody ELISA.

Study Day 450 (3 months +/-14 days after Third Vaccination)
1. Perform basic history and physical exam, emphasizing examination of any acute complaints.
2. Obtain approximately 5 mL of blood for hematology analysis and filter paper collection.

Study Day 480 (4 months +/-14 days after Third Vaccination)
1. Perform basic history and physical exam, emphasizing examination of any acute complaints.
2. Obtain approximately 10 mL of blood for hematology, filter paper, and anti-AMA1 antibody ELISA.

Study Day 510 (5 months +/-14 days after Third Vaccination)

1. Perform basic history and physical exam, emphasizing examination of any acute complaints.
2. Obtain approximately 5 mL of blood for hematology analysis and filter paper collection.

Study Day 540 (6 months +/-14 days after Third Vaccination)

1. Perform basic history and physical exam, emphasizing examination of any acute complaints.
2. Obtain approximately 35 mL of blood for hematology, anti-AMA1 antibody ELISA, B cell analysis, filter paper collection, and GIA.

7.7 Laboratory Testing

Using standard techniques, the MRTC Clinical Laboratory of the DEAP/FMPOS will perform the following tests. Tests will be performed either on-site in Donéguébougou or at the FMPOS campus in Bamako.

1. Complete blood count plus partial white blood cell differential (granulocyte count, lymphocyte count, and mononuclear cell count)
2. Serum creatinine
3. Alanine aminotransferase (ALT)
4. HBsAg ELISA
5. HCV ELISA

Urine β-hCG testing will be performed at the clinical trial site using an U.S. Food and Drug Administration (FDA)-approved urine pregnancy test kit. Urine dipstick testing will be performed at the trial site using an FDA-approved product.

Anti-AMA1 ELISAs will be performed both at the MVDB in Rockville, Maryland, and at the MRTC in Bamako, Mali. Only AMA1 ELISA results obtained at the MVDB will be used for the analysis of vaccine-induced immunity; assays will also be performed at the MRTC as part of an on-going transfer of technology and techniques. Results obtained in Mali will be compared to those obtained in the US only after the results from the MVDB have been entered into the database and the database has been locked.

GIAs will be performed at the MVDB in Rockville, Maryland. B cell analyses will be performed at the MRTC/DEAP in Bamako, Mali, and/or the MVDB in Rockville, Maryland.

7.8 Immunologic Testing

7.8.1 Antibody Assay

Antibody levels to the AMA1 antigens will be measured in serum by ELISA. Duplicate assays will be done for both 3D7 and FVO. Briefly, microwell plates are coated with antigen solution. Plates are washed with TRIS-buffered saline (TBS) containing Tween-20 (T-TBS) and blocked with TBS containing skim milk powder. After washing

with T-TBS, diluted serum samples are added in triplicate and incubated at room temperature. After incubation, unbound antibodies are removed by washing the plates with T-TBS, and alkaline phosphatase-conjugated goat anti-human IgG solution is added to each well and incubated for 2 hours at room temperature. Plates are then washed with T-TBS, followed by adding phosphatase substrate solution to each well; the plates are then covered and incubated for 20 minutes at room temperature for color development. The plates are read immediately at 405 nm with a microplate reader. The optical density values are used to determine antibody concentration for AMA1 by comparing to a standard curve formulated with known positive control sera included on each ELISA plate.

7.8.2 Growth Inhibition Assay

The GIA is designed to determine whether anti-AMA1 antibodies obtained from an immunized animal or person can inhibit the process of merozoite invasion into red cells. In this assay, synchronized blood-stage parasites are incubated with sera from volunteers for a period of 40 hours *in vitro*. During this period, merozoites emerge from the infected red cells, invade normal red cells, and initiate a new growth cycle. Parasite growth and development in the newly invaded red cells are assessed in our studies by quantitating the activity of a parasite metabolic enzyme—lactate dehydrogenase. Enzyme activity determined by a colorimetric assay is proportional to the number of parasites. Results with immune sera are compared to results with normal nonimmune sera and then expressed as percent inhibition of parasites.

7.8.3 B cell analysis

Studies of the B cell response to vaccination will be purely exploratory and will not be considered an endpoint for the purposes of analysis. Fluorescently labeled antibodies to different immune cell markers (e.g., CD19, CD27, and CD38) will be used to characterize, by multiparameter flow cytometry, the B cells from the peripheral blood of volunteers enrolled in the study, both before and after vaccination. The B cells specific for the vaccine antigen will be identified using fluorescently labeled AMA-1 proteins in flow cytometry. In addition, an ELISPOT assay may be used to access antigen-specific antibody production on an individual cell basis.

Although presently in the planning stage, additional studies such as single cell PCR analysis of immunoglobulin heavy and light chain to assess immunoglobulin repertoire and mutational status, as well as microarray and immunoaffinity capillary electrophoresis for analysis of genes and proteins that are not able to be detected by multiparameter flow cytometry, may be performed as exploratory studies of the B cell response to vaccination in persons repeatedly exposed to malaria.

7.8.4 Plasmodium falciparum genotyping

Venous blood from study participants will be spotted onto filter papers at regular intervals throughout the study (see **Appendix D**). These will be stored for future *P. falciparum* DNA extraction and sequencing of parasite gene polymorphisms. These stored samples will NOT be used to assess the efficacy of the AMA1-C1/Alhydrogel®

vaccine. Rather, the information obtained from the molecular analysis of *P. falciparum* infections in immunized individuals could prove invaluable in designing future malaria vaccines, should the AMA1-C1/Alhydrogel® vaccine eventually prove less than 100% effective.

8.0 ADVERSE EVENTS MONITORING AND REPORTING

8.1 Definitions

8.1.1 Adverse Event

An adverse event (AE) includes any noxious, pathological or unintended change in anatomical, physiological or metabolic functions as indicated by physical signs, symptoms and/or laboratory-detected changes occurring in any phase of the clinical study, whether associated with the study vaccine or active comparator, and whether or not considered vaccination related. This includes an exacerbation of pre-existing conditions and intercurrent illnesses. All AEs must be graded for intensity and relationship to the investigational vaccine as described in **Section 8.2.2** and **Section 8.2.3** in this protocol.

8.1.2 Serious Adverse Event (SAE)

An SAE is an AE, whether considered related to the investigational vaccine or not, meeting one of the following conditions:

1. Death during the period of protocol-defined surveillance
2. Life threatening: defined as an event that places a subject at immediate risk of death at the time of the event and does not refer to an event that hypothetically might have caused death were it more severe
3. Hospitalization during the period of protocol-defined surveillance: defined as at least an overnight stay in the hospital or emergency ward for treatment that would have been inappropriate if administered in the outpatient setting
4. Results in a congenital anomaly or birth defect
5. Results in a persistent or significant disability or incapacity: defined as a substantial disruption of the study participant's ability to carry out normal life functions
6. Any other important medical event that may not result in death, be life threatening, or require hospitalization, may be considered a serious AE when, based upon appropriate medical judgment, the event may jeopardize the subject and may require medical or surgical intervention to prevent one of the outcomes listed above

8.2 Assessment of Adverse Events

8.2.1 Identification of AEs

Assessment of safety will include clinical observation and monitoring of hematological, chemical, and immunologic parameters. Safety will be evaluated by monitoring of volunteers for local and systemic adverse reactions during the course of the trial. Volunteers will be closely monitored for 30 minutes following each immunization.

Additionally, volunteers will return to the clinic on Days 1, 2, 3, 7, and 14 following each vaccination for clinical assessments.

All AEs will be graded for intensity and relationship to study product. Reactions will be graded as described in **Section 8.2.2** in this protocol. A study clinician will be available 24 hours a day during the study evaluation period; a study clinician will stay in Donéguébougou for the duration of the trial and will be available to the study participants at all times. Should a volunteer call on a study clinician to report an adverse event, it will be fully documented in the volunteer's study chart, and discussed with the Principal Investigator.

8.2.2 Determination of Severity

All AEs will be assessed by the investigator using the following protocol-defined grading system:

Grade 0 (None)	
Grade 1 (Mild):	No effect on activities of daily living
Grade 2 (Moderate):	Partial limitation in activities of daily living (can complete ≥ 50% of baseline), or treatment given
Grade 3 (Severe):	Activities of daily living limited to < 50% of baseline, or medical evaluation required
Grade 4:	Serious Adverse Event (see **Section 8.1.2**)

Intensity of the following AEs will be assessed by the investigator as described in **Table 7**. All laboratory AEs will be graded in severity following the toxicity table in **Appendix E**.

Table 7 Assessment of Adverse Event Intensity

AdverseEvent	Grade	Intensity
Pain at injection site	0	Absent
	1	Pain that is easily tolerated
	2	Pain that interferes with daily activity
	3	Pain that prevents daily activity
Erythema at injection site	0	0 mm
	1	>0 - ≤20 mm
	2	>20 - ≤50 mm
	3	>50 mm
Swelling at injection site	0	0 mm
	1	>0 - ≤20 mm
	2	>20 - ≤50 mm
	3	>50 mm
Fever (oral)	0	≤37.5°C
	1	37.6°C - 38.0°C
	2	>38.0°C – 39.0°C
	3	>39.0°C
Headache	0	None
	1	Headache that is easily tolerated
	2	Headache that interferes with daily activity
	3	Headache that prevents daily activity
Nausea	0	None
	1	Nausea that is easily tolerated
	2	Nausea that interferes with daily activity
	3	Nausea that prevents daily activity
Malaise	0	None
	1	Malaise that is easily tolerated
	2	Malaise that interferes with daily activity
	3	Malaise that prevents daily activity
Myalgia	0	None
	1	Myalgia that is easily tolerated
	2	Myalgia that interferes with daily activity
	3	Myalgia that prevents daily activity
Arthralgia	0	None
	1	Joint pain that is easily tolerated
	2	Joint pain that interferes with daily activity
	3	Joint pain that prevents daily activity
Urticaria	0	None
	1	Requiring no medications
	2	Requiring PO or topical treatment or IV medication or steroids for <24 hours
	3	Requiring IV medication or steroids for >24 hours

8.2.3 Association with Receipt of the Study Vaccine

All AEs will have their possible relationship to study vaccine assessed using the following terms:

<u>Definite</u>: Clear-cut temporal association, and no other possible cause.

<u>Probable</u>: Clear-cut temporal association and a potential alternative etiology is not apparent.

Possible: Less clear temporal association; other etiologies also possible.

Remote: Temporal association between the AE and the vaccine or the nature of the event is such that the vaccine is not likely to have had any reasonable association with the observed illness/event (cause and effect relationship improbable but not impossible).

Not Related: The AE is completely independent of vaccine administration; and/or evidence exists that the event is definitely related to another etiology.

The degree of certainty with which an AE can be attributed to administration of the study vaccine will be determined by how well the event can be understood in terms of one or more of the following:

1. The event being temporally related with vaccination or reproduced on re-vaccination.
2. A reaction of similar nature having previously been observed with this type of vaccine and/or formulation.
3. The event having often been reported in the literature for similar types of vaccines.

All local (injection-site) reactions will be considered causally related to vaccination.

8.3 Adverse Event Reporting

All SAEs will be reviewed by a study physician, recorded on the appropriate SAE form, and followed through to resolution by a study physician. All SAEs will be reported by email, telephone or fax within 1 working day of notification of the SAE occurrence to the PI, to all of the following:

- Sponsor (Regulatory Compliance and Human Subjects Protection Branch [RCHSPB]/NIAID): Phone: 301-846-5301, Fax: 301-846-6224
- FMPOS Comité d'éthique (CE): Phone: (223) 222-5277
- NIAID Institutional Review Board (IRB): Phone: 301-435-9273, Fax: 301-435-6739
- NIAID Data, Safety, and Monitoring Board (DSMB): Phone:301-846-6553, Fax: (301) 846-6224

Following notification from the investigator, RCHSPB as the Investigational New Drug (IND) sponsor, will report events that are both serious and unexpected that are possibly, probably, or definitely related to the vaccine, to the FDA within the required timelines: fatal and life-threatening events within 7 calendar days (by phone or fax) and all other SAEs in writing within 15 calendar days. All SAEs not listed as possibly, probably, or definitely related will be reported to the FDA at least annually in a summary format.

All local and systemic reactions not meeting the criteria for "serious adverse events" will be captured on the appropriate case report form (CRF). These events will be followed to resolution. Grade 3 adverse events deemed definitely or probably related to vaccination will be reported by email or fax within 15 working days of the PI becoming aware of the event, to the Sponsor, the NIAID IRB, the FMPOS ethics committee, and the NIAID DSMB.

8.4 Adverse Event Monitoring

8.4.1 Medical Monitor

An independent medical monitor, Professor Aly Guindo, has been appointed for oversight of participant safety in this trial. The Medical Monitor will be available to advise the investigators on trial-related medical questions or problems. Should Pr. Guindo not be available, Dr. Kalifa Sangaré, the district health officer for the Donéguébougou area, will serve as a substitute independent Medical Monitor.

The Medical Monitor's primary responsibility will be to monitor participant safety. The Principal Investigator is responsible for ensuring that the Medical Monitor is aware of any new safety information that becomes available during the course of the trial.

8.4.2 Data Safety and Monitoring Board (DSMB)

Because this is a randomized and blinded study, NIAID policy mandates that it be reviewed by the permanent NIAID DSMB. This DSMB has been constituted to review the data and analysis plans of all intramural NIAID clinical studies that require DSMB oversight, and consists of experts in infectious diseases, biostatistics, and clinical trials. In addition, the local Medical Monitor may be added as an ad hoc member for the duration of the study, at the discretion of the DSMB. The DSMB will serve in an advisory capacity to the investigators, the sponsor, and the FMPOS and NIAID IRBs, which will consider its recommendations seriously in deciding whether or not the study may proceed. The Board meets at regular periods during the year, but has been empowered to convene in person or via teleconference between their regularly scheduled meetings should the need arise.

This protocol will be submitted to the DSMB for their review. All cumulative safety data reports from the trial will be submitted to the Board before each dose escalation (i.e., between the first dose in Cohort 1 and Cohort 2, between the first dose in Cohort 2 and Cohort 3, between the second dose in Cohort 1 and the second dose in Cohort 2, and so on). Safety data reports will include data from at least the first seven days after vaccination. After the third and final vaccination has been administered to all three dose cohorts, additional safety and immunology results and reports will be submitted to the DSMB as they become available. A final report will be submitted to the DSMB following completion of the study.

The DSMB will review cumulative safety data for evidence of study-related AEs, adherence to the protocol, and factors that may affect outcome or study data such as protocol violations and losses to follow-up. Conference calls between the investigators and the DSMB will be scheduled within the week prior to each dose escalation (i.e., after the first dose is administered to Cohort 1 but before the first dose is administered to Cohort 2, etc.). If no criteria for suspending the study are met (see **Section 8.5**), dose escalation will proceed with approval from the DSMB. Should the DSMB not be able to meet, either in person or via teleconference, prior to scheduled dose escalation, the safety reports may be distributed to individual DSMB members who will then submit their vote for or against dose escalation; the DSMB chair will tally the votes and communicate the

collective decision to the Principal Investigator. If none of these options are possible, the DSMB may reserve the right to defer to the independent Medical Monitor to decide whether or not escalation may proceed. If a deferral to the Medical Monitor occurs, the IRBs will be promptly notified.

Written approval (via letter or email) to proceed to the next dose of vaccine must be obtained from the DSMB (or Medical Monitor in the event that the DSMB cannot review the safety data in a timely manner, as described above) prior to administration. Both the DSMB and Medical Monitor will have access to the randomization code, as they may wish to review the data in an unblinded fashion should significant safety questions arise prior to the final unblinding.

It is the Principal Investigators'(or designated agent) responsibility to ensure that the DSMB reviews the current safety data (grouped by dose cohort), study protocol, and any other requested documents at its meetings. Occurrence of an SAE will be reported to the DSMB at the same time that it is reported to the IRBs. Additionally, any new information that may adversely affect the safety of the subjects or the conduct of the study will be submitted to the DSMB as it becomes available.

8.5 Criteria for Placing the Study on Hold

If a dose of vaccine is considered significantly reactogenic (see below), dose escalation and/or additional vaccinations will be suspended until reviewed by the Medical Monitor, DSMB and study sponsor (RCHSPB). The communications from the DSMB will subsequently be forwarded by the investigators to the FMPOS and NIAID IRBs.

The following criteria will be used to define significant reactogenicity (note that these apply to both the AMA1-C1/Alhydrogel® and Recombivax HB® vaccines, as these criteria will be applied in advance of any consideration of unblinding):

1. One or more volunteers experience a serious AE (as defined in **Section 8.1.2** in this protocol) that is determined to be possibly, probably, or definitely related to the vaccine (as defined in **Section 8.2.3** in this protocol), **OR**
2. One or more volunteers experience anaphylaxis that is probably or definitely related to the vaccine, **OR**
3. One or more volunteers in a single dose cohort experience an objective physical finding or laboratory abnormality of Grade 3 or higher (with the exception of isolated Grade 3 erythema or swelling), as defined in **Section 8.2.2** in this protocol, that is determined to be, probably, or definitely related to the vaccine.

8.6 Breaking the Study Blind

As outlined in **Section 4.5**, the study will double-blinded for the first two immunizations and until all study participants have had their Day 180 visit, after which the investigators will be unblinded. During the double-blinded part of the study, a study participant's randomization code may be unblinded only for safety purposes. This is very unlikely to occur, as once a vaccine is administered, knowing which vaccine was given is unlikely to influence the medical management of an adverse event. This procedure is therefore

exceptional and any decision to unblind will be discussed with the sponsor, the PI, the Scientific Investigators, the Medical Monitor, and the DSMB. If deemed necessary for urgent safety reasons, the Medical Monitor, in consultation with the DSMB (if possible in a timely manner), may unblind a specific participant without revealing the study blind to the investigators and the sponsor. Any opening of the envelope containing the randomization code will be thoroughly documented. It is to be emphasized that the Medical Monitor may put the study on hold at any time and discuss with the DSMB. The decision to completely unblind or permanently stop the study prior to Day 180 will take the final form of a formal recommendation by the DSMB to the study sponsor. The PI must then notify the IRBs of this decision.

In the event that the investigators come to know the study code prior to final unblinding, the PI must notify the sponsor immediately. The reasons will be documented by the PI and added to the study file.

Unblinding of the investigators will be done after all study participants have completed the Day 180 visit. This will take place only after monitoring and verification of cGCP compliance by RCHSPB, DSMB review to verify all safety concerns have been addressed, and after all safety and immunological results have been entered and the databases locked. The final decision to unblind will be made jointly by the sponsor, the investigators, and the DSMB.

9.0 DATA COLLECTION AND MONITORING

9.1 Source Documentation

CRFs will be used to record data for subjects enrolled in the study. In addition, supplementary documents (laboratory test reports, supplementary hospital or medical records, etc.) may form part of the source documentation for a study participant. The Investigator is responsible for the accuracy and completeness of the data reported in the CRFs and the source documents. Data reported in the CRFs that is derived from source documents should be consistent with source documents and the discrepancies should be explained.

9.2 Study Documentation

Study-related documentation will be completed as required by the IRBs, the sponsor, and regulatory authorities. Continuing review documentation will be submitted by the Investigator to the IRBs on the anniversary date of initial review as specified by each IRB. An annual report will be submitted by the sponsor to the FDA on the anniversary date that the IND for AMA1-C1/Alhydrogel® malaria vaccine went into effect. These reports will provide a brief description of the progress of the investigation as outlined in 21 *Code of Federal Regulations* 312.33, and will include any revisions of the protocol.

The investigators will maintain adequate records of the disposition of the investigational product, including dates, quantity, and use by subjects. If the study is terminated, suspended, or completed, the investigators will return all unused supplies of the investigational product to the sponsor.

9.2.1 Study Reports

In addition to the study-related documentation required by the regulatory authorities, the MVDB will also submit two reports to the sponsor. The first, or interim, report will be completed after the safety and immunogenicity data (excluding the B cell analyses) from the 6-month visit has been compiled and the study investigators have been unblinded. This interim report will therefore contain all information obtained up to study Day 180 of the third cohort (i.e., after all three cohorts have received their second dose of vaccine, as well as the information obtained during the extended follow-up during the 2004 malaria transmission season). This report will serve as the basis for deciding whether to continue with future Phase 1 testing of the formulation in malaria-exposed children, which is presently being planned for Mali in the spring of 2005.

The interim report will present safety and immunogenicity results by individual volunteer, dose cohort, and vaccine allocation (AMA1-C1/Alhydrogel® vs. Recombivax HB®). This report will be submitted to both the sponsor and the DSMB. Based on this report, the MVDB, in consultation with the sponsor and DSMB, will decide whether or not dose de-escalation to children may proceed.

A final report will be submitted by the investigators to the sponsor after trial completion. This final report will therefore contain the safety and immunogenicity data obtained after the third and final immunization, including the extended follow-up through to the end of the 2005 malaria transmission season.

9.3 Retention of Records

Trial-related documents will be maintained by the Investigator for a period of 2 years after final marketing approval of the vaccine, or if 2 years have elapsed since the formal discontinuation of clinical development of the product. The sponsor is required to inform the PI as to when such documents need no longer be retained. Storage of all trial-related documents will be such that confidentiality will be strictly maintained.

9.4 Protocol Revisions

No revisions to this protocol will be permitted without documented approval from both the sponsor and the IRBs that granted the original approval for the study. This does not apply to changes made to reduce discomfort or avert risk to study volunteers. Furthermore, in the event of a medical emergency, the investigators shall perform any medical procedures that are deemed medically appropriate. The PI must notify the sponsor of all such occurrences. Any change to the protocol will be submitted to the participating IRBs (NIAID and FMPOS) as a protocol amendment, and changes not affecting risk to volunteers may be expedited, as appropriate.

9.5 Clinical Investigator's Brochure

Investigators will receive the current version of the Clinical Investigator's Brochure, which comprehensively describes all the available preclinical and human experience with the experimental vaccine. If relevant new information becomes available during the

course of the trial, the investigators will receive a revised Investigator's Brochure or an amendment to the current version.

9.6 Study Monitoring

The sponsor (RCHSPB or its designee) and/or World Health Organization (WHO) will monitor all aspects of the study, with respect to current Good Clinical Practices, for compliance with applicable government regulations. Prior to the start of the study, the PI will be informed of the frequency of monitoring visits and will be given reasonable notification prior to each visit. The objectives of a monitoring visit will be to verify the prompt reporting of SAEs, to check the availability of the signed Informed Consent for enrolled study participants, and to compare CRFs and spreadsheets with source data for completeness and accuracy. During the monitoring visit, the PI (and/or designee) and other study personnel should be available to discuss the study. Study documents must be available for review throughout the course of the study. The sponsor will retain originals of the FDA Form 1572 and copies of other study documents as deemed necessary.

10.0 STATISTICAL CONSIDERATIONS

10.1 General Design

The goal of this Phase 1 vaccine trial is to assess the safety and immunogenicity of AMA1-C1/Alhydrogel® malaria vaccine in human volunteers. The purpose of the Phase 1 trial is to optimize a candidate asexual blood-stage vaccine for dose and schedule, as measured by low reactogenicity, immune response, and persistence of antibody.

10.1.1 Description of the Statistical Methods to Be Employed

The purpose of this trial is to estimate adverse event rates and patterns of immune response as well as to compare these rates and patterns between the investigational and comparator vaccines, in different doses of the study vaccine, and to compare the response by clone.

This section briefly describes the statistical methods to be used; a detailed analytic plan will fully describe the methods. The analytic plan will discuss the planned approaches to missing data. Listings will show all observed data and, if applicable, imputed values and the approaches taken for imputation. Deviations from the original analytic plan will be thoroughly documented and reported to the sponsor.

Estimates will be presented with their 90% confidence intervals. Descriptive approaches will be used to meet most of the objectives of the protocol stated in **Section 2.0**. Formal statistical tests will be used to compare doses and clones. Results will be presented in tables and graphs.

Because of the small sample size of this study, statistical tests will be performed without correction for multiplicity. A nominal Type I error rate of 10 percent will be used.

Most of the analyses of immunogenicity will be based on a longitudinal mixed model with terms for dose group and clone. The detailed analysis plan will describe the method of modeling and the approach to selecting a covariance structure.

Primary Objective: To estimate the frequency of vaccine-related AEs, graded by severity, for each dose.

a. The frequency of immediate, systemic, and local AEs will be summarized.
b. A line listing of each clinical and laboratory AE classified as immediate (within the first 30 minutes), systemic, and local will be displayed in tables stratified by vaccine allocation and dose cohort.
c. Episodes of clinical malaria will be counted as AEs and the frequency compared between groups, by transmission season. To ensure that lack of observed clinical malaria is not due to lack of exposure and infection, groups will be compared as to whether or not at least 25% were parasitemic (as determined by PCR of filter paper samples) at any time point during each of the two transmission seasons.
d. AEs will be summarized by severity and relationship to vaccine by individuals and dose cohort.

The proportion of volunteers with at least one local adverse event will be compared by dose cohort, and tests performed to assess whether the four cohorts differ with respect to these proportions and whether there is a dose-response relationship in the four cohorts.

Secondary Objective 1: To determine the dose that generates the highest antibody concentration at Day 42, as judged by enzyme-linked immunosorbent assay (ELISA).

Anti-AMA1 antibody will be measured by ELISA on Days 0, 14, 28, 42, 90, 150, 180, 270, 360, 374, 420, 480, and 540. To exploit the multiple measures of antibody within each subject, a longitudinal model with an appropriate covariance structure will be built to describe the antibody response over time. The model will include all four dose cohorts (Recombivax HB®, 5 µg, 20 µg, and 80 µg) and a term for the AMA1 FVO or AMA1 3D7 clone. The level of antibody at Day 42 will be estimated from the resulting model collapsing over the two clones. Formal statistical tests will assess whether the response is monotone (Recombivax HB® < low dose < medium dose < high dose) for the four dose cohorts.

Various exploratory methods will be used to assess the sensitivity of the results to the assumptions in the model.

Secondary Objective 2: To assess and compare the duration of antibody response to AMA1 FVO and AMA1 3D7 by ELISA.

The model described above will be used to estimate antibody response over time, by dose, and to compare the patterns for AMA1 FVO and AMA1 3D7.

Secondary Objective 3: To measure the inhibition of parasite growth as measured by the in vitro GIA to both FVO and 3D7 parasite clones.

Graphs will display growth inhibition expressed as a percent of inhibition comparing test sera to preimmune sera. Depending on the distribution of the data, parametric or non-parametric methods will be used to compare inhibition as a function of dose, and clone.

Secondary Objective 4: To determine the relationship between anti-AMA1 antibody concentration, as judged by ELISA, and degree of in vitro growth inhibition of *P. falciparum* in a GIA.

A non-linear curve will be fit to determine the goodness of fit of the data to a hyperbolic function and to determine the ELISA value giving 50 percent inhibition.

Secondary Objective 5: To determine the relative specificity of the antibodies to a range of AMA1 serotypes, in addition to FVO and 3D7, as judged by ELISA, and growth inhibition on a select panel of parasites with typed AMA1.

If enough data are available, exploratory analyses will examine a range of AMA1 clones.

Should the study be terminated early (see **Section 8.5**), the investigative team will discuss with the DSMB the reason for termination and determine which study questions can be addressed in an unbiased manner with the available data. The available data will be analyzed and interpreted in light of early termination.

10.1.2 Safety

The primary safety endpoint is the frequency of vaccine-related AEs, as classified by both intensity and severity through active and passive surveillance. Separate assessments of systemic and local reactions will be performed. Comparisons will be made between the AMA1-C1/Alhydrogel® and Recombivax HB® vaccines.

10.1.3 Immunogenicity Analysis

One primary immunogenicity endpoint will be evaluated. Anti-AMA1 antibody will be measured by ELISA on Days 0, 14, 28, 42, 90, 150, 180, 270, 360, 374, 420, 480, and 540, as listed in the schedule of visits (see **Appendix D** in this protocol).

10.2 Sample Size

Based on an analysis of the human antibody responses to a number of malaria antigens that have been tested in clinical trials (the three components of Combination B [34] and RTS,S [35]), the observed coefficient of variation in the range of antibody concentrations has been found to be remarkably constant at approximately 1.2 - 1.4. Based on the distribution of antibody responses for each of the four antigens in these trials, a sample size of 10 per dose group will permit detection of at least a fivefold difference in antibody concentration between groups using a Mann-Whitney test, assuming a level of significance of 0.05 and a power of 0.80. Because the actual analyses to be used will be

more efficient than a Mann-Whitney test, the power for detecting a fivefold difference is expected to be higher than 0.80. A group size of 10 volunteers per dose gives a probability of 0.80 for detecting one or more serious or severe AE that occurred with a probability of 0.15 per volunteer. Given the length of this study and the possibility of volunteer drop-out, an extra 2 volunteers will be added to each group.

Since comparisons will be made between three different dose concentrations of AMA1-C1, three groups of 12 volunteers each will receive this vaccine. In addition, each dose concentration of AMA-1C1 will be compared to the control vaccine, which will serve as both a background for immunologic assessments and a safety comparison. This control group will receive Recombivax HB®, and should also contain at least 10 volunteers. To enable ease of randomization, we have decided to enroll a total of 18 controls, 6 per each dose concentration of AMA1-C1; thus, each group will contain 12 volunteers receiving AMA1-C1 plus 6 receiving Recombivax HB® (2:1 distribution). The total sample size will therefore be 54.

11.0 PROTECTION OF HUMAN SUBJECTS

11.1 Institutional Review Board/Ethics Committee

The investigators will be responsible for obtaining IRB approvals for the study. Before the start of the study, the appropriate documents (including the protocol, Investigator's Brochure, and informed consent form) will be submitted to the IRBs. A copy of the study approval (including approval of the informed consent form) is to be maintained in the study document binder and a copy will be supplied to the sponsor. During the study, the PI is responsible for providing the IRBs with all documents subject to review (i.e., protocol amendments, informed consent form updates, and any written information that may be provided to the subject). Annual reports on the progress of the study will be made to the IRBs by the investigators in accordance with IRB guidelines and government regulations.

11.2 Informed Consent

In obtaining and documenting informed consent, the Investigator must comply with the applicable regulatory requirements, current Good Clinical Practices, and ethical principles. The written informed consent form must be approved by all IRBs prior to its use.

11.3 Risks

Risks to the volunteers are associated with venipuncture and with immunization. These risks are outlined below.

Female participants will be cautioned of the unknown risk of the AMA1-C1 vaccine to the fetus and will be advised to use adequate birth control methods until one month following the third immunization. Any female participant interested in contraceptive methods will be referred to the local health center family planning services for evaluation and institution of an appropriate contraceptive method.

11.3.1 Venipuncture

Risks occasionally associated with venipuncture include pain and bruising at the site of venipuncture, lightheadedness, and syncope (rarely).

11.3.2 Immunization with AMA1-C1/Alhydrogel®

Possible local vaccine reactions include pain, swelling, erythema, induration, limitation of limb movement for several days, lymphadenopathy, or pruritus at the injection site. Local subcutaneous nodules, believed to be granulomatous reactions to aluminum hydroxide, have been observed with use of aluminum hydroxide-based adjuvants. Thus, most aluminum hydroxide-adsorbed vaccines are injected intramuscularly rather than subcutaneously. Systemic reactions such as fever, chills, headache, fatigue, malaise, myalgia, joint pain, may also possibly occur. Immediate hypersensitivity reactions including urticaria, anaphylaxis, or other IgE-mediated responses are possible as with any vaccine. As with any investigational vaccine, there is a theoretical possibility of risks about which we have no present knowledge. Volunteers will be informed of any such risks should further data become available.

Additionally, a hypothetical safety concern exists due to the fact that volunteers in this study will be challenged with naturally-occurring infection shortly after receiving the first two doses of vaccine (i.e., the malaria transmission season will start soon after the second vaccination has been administered). Accordingly, volunteers will be followed at several timepoints throughout the 2004 malaria transmission season. Furthermore, volunteers will be followed throughout the 2005 malaria transmission season, after the third and final vaccination is administered to all volunteers on study Day 360.

11.3.3 Immunization with Recombivax HB®

Adverse reactions may be local and include redness, warmth, edema, induration with or without tenderness as well as urticaria, and ecchymosis [33]. Malaise, transient fever, hypotension, nausea, diarrhea, headache, and arthralgia may develop in some patients after the injection. An apparent hypersensitivity syndrome (serum-sickness-like) of delayed onset has been reported days to weeks after vaccination, including: arthralgia/arthritis (usually transient), fever, and dermatologic reactions such as urticaria, erythema multiforme, ecchymoses and erythema nodosum.On rare occasion, non-fatal anaphylaxis has been reported following administration of Recombivax HB®.

11.4 Precautions taken to Minimize Risks

11.4.1 Immunization

As outlined above, the participants will be monitored closely during their participation in this study. The study vaccines have been produced according to Good Manufacturing Procedures (GMP). The vaccines will be administered by experienced investigators with drugs and equipment available for the treatment of anaphylaxis and other potential adverse reactions. All vaccine doses will be given by intramuscular injection to minimize injection site reactions such as pain.

11.4.2 Protection of Study Staff

All study personnel have been trained to follow Universal Precautions. Additionally, the following approved Standard Operating Procedures from the MRTC clinical lab elaborate the precautions that will be taken by study personnel to minimize risks: General Laboratory Safety, Exposure to Blood and Infectious Material, and Waste Management. The procedures for handling exposures to blood and infectious material are in keeping with the rules and regulations of the local Malian authorities, including the Malian initiative addressing access to antiretroviral medications.

11.5 Benefits

Volunteers may not receive any direct benefit from participation in this study. It is hoped that information gained in this study will contribute to the development of a safe and effective malaria vaccine. Volunteers who receive Recombivax HB® during the course of the study proper may receive the benefit of subsequent protection against exposure to the hepatitis B virus. Volunteers who receive the AMA1-C1/Alhydrogel® vaccine during the trial will be offered Recombivax HB® after the conclusion of the study, and thus may also receive this benefit. This will be administered at the recommended schedule of 0, 1, and 6 months.

Free medical treatment will be provided to all <u>enrolled</u> participants during the active immunization phase and the follow-up period. The pharmacy at the clinic will have sufficient provisions to provide participants with drugs for the treatment of minor illnesses free of charge. If further evaluation or treatment is necessary the participant will be referred one of the National Hospitals in Bamako or Kati located within 40 km of Donéguébougou. If the investigators judge that a participant requires hospitalization in Bamako or Kati, referral and transportation to these places will be arranged and the medical management of the participant will be monitored by a senior physician-investigators and the local Medical Monitor. Medical care for ailments not related to vaccination will not extend beyond the study period. Medical care for ailments related to vaccination will extend at least until the condition has resolved.

In practice, the MRTC-run clinic in Donéguébougou has been the primary provider of medical care in Donéguébougou since 1995. This clinic provides care free-of-charge; hence the entire community – and not just study participants – benefits from its presence.

Study participants who develop clinical malaria during the course of the study will be treated according to the guidelines of the Malian National Malaria Control Program. All medications used for treatment are licensed in Mali, and have proven safety records.

11.6 Confidentiality

All study-related information will be stored securely at the study site in Donéguébougou or at the MRTC offices in Bamako, Mali. All participant information will be stored in locked file cabinets in areas with access limited to study staff. All laboratory specimens, reports, study data collection, process, and administrative forms will be identified by

coded number only to maintain participant confidentiality. All computer entry will be done by coded number only, and all local databases will be secured with password-protected access systems. Forms, lists, logbooks, appointment books, and any other listings that link participant ID numbers to other identifying information will be stored in a separate, locked file in an area with limited access.

Participants' study information will not be released without the written permission of the participant, except as necessary for monitoring by NIAID and/or its contractors, the FDA, and the WHO.

11.7 Compensation

Volunteers will not be given any monetary compensation. Volunteers will be given 75 kg of rice and 75 kg millet, in three equal installments, to compensate for the time taken to come to the study clinic for study-related visits. The amount of cereal has been determined after consultation with village elders, and represents an equivalent for the amount of time taken from working in their fields (total value of approximately USD$90). The first installment will be distributed after all participants have received the first injection, the second will be distributed after Day 180, and the third will be given at the conclusion of the study.

Throughout Mali, the availability of food is subject to seasonal variation in relation to the harvest season. However, there is no recent history of famine or starvation. In the region of our study site, while cases of pediatric malnutrition are occasionally seen at the village health clinic, these are attributable to poor feeding habits rather than to scarcity of food, and the intervention is to educate parents to provide more nutritional foods to small children. The total amount of food to be distributed in three parts over the course of one year will last an average family approximately four weeks. The type of food distributed – rice and millet – are staple starches that are typically served accompanied by a sauce containing some sort of meat as well as vegetables, and therefore are only a part of the local diet. This amount of compensation is consistent with what we have provided to participants of longitudinal studies in Mali for several years, and has been carefully considered by the local Malian IRB, who have determined that it is appropriate compensation for time lost to study procedures, and is not coercive.

12.0 REFERENCES

1. UNDP/World Bank/WHO Special Programme for Research and Training in Tropical Diseases (TDR). *TDR Twelfth Program Report*. 57-76. 1997.
2. Diani F. Evaluation de la situation sanitaire au Mali. Thèse de Pharmacie. Université de Bamako, Bamako, Mali (1985).
3. Programme National de Lutte Contre le Paludisme. Politique Nationale de Lutte Contre le Paludisme au Mali. Deuxième revision. Bamako, Mali (2003).
4. Niambele, M.B. Thèse de Medicine. Université de Bamako, Bamako, Mali (1999).
5. Poudiougou, B., Cisse, M.B., Ranque, S., et al. Risk Factors of Severe and Complicated Malaria and Malaria Specific Mortality in Pediatric Hospital in Bamako, Mali. *Am. J. Trop. Med. Hyg.* 69(3) Suppl:305-6 (2003).

6. Kone, A., Dicko, A., Coulibaly, D., et al. Clinical Presentation and Risk Factors for Death from Severe Falciparum Malaria in Bandiagara, Mali. *Am. J. Trop. Med. Hyg.* 69(3) Suppl:317-8 (2003).
7. Good, M.F., Kaslow, D.C. & Miller, L.H. Pathways and strategies for developing a malaria blood-stage vaccine. *Annu. Rev. Immunol.* 16, 57-87 (1998).
8. Narum, D.L. & Thomas, A.W. Differential localization of full-length and processed forms of PF83/AMA1 an apical membrane antigen of *Plasmodium falciparum* merozoites. *Mol. Biochem. Parasitol.* 67, 59-68 (1994).
9. Crewther, P.E., Culvenor, J.G., Silva, A., Cooper, J.A. & Anders, R.F. *Plasmodium falciparum*: two antigens of similar size are located in different compartments of the rhoptry. *Exp. Parasitol.* 70, 193-206 (1990).
10. Waters, A.P. et al. A merozoite receptor protein from *Plasmodium knowlesi* is highly conserved and distributed throughout *Plasmodium*. *J. Biol. Chem.* 265, 17974-17979 (1990).
11. Peterson, M.G. et al. Integral membrane protein located in the apical complex of *Plasmodium falciparum*. *Mol. Cell Biol.* 9, 3151-3154 (1989).
12. Kocken, C.H. et al. Precise timing of expression of a *Plasmodium falciparum*-derived transgene in *Plasmodium berghei* is a critical determinant of subsequent subcellular localization. *J. Biol. Chem.* 273, 15119-15124 (1998).
13. Deans, J.A. et al. Rat monoclonal antibodies which inhibit the in vitro multiplication of *Plasmodium knowlesi*. *Clin. Exp. Immunol.* 49, 297-309 (1982).
14. Thomas, A.W., Deans, J.A., Mitchell, G.H., Alderson, T. & Cohen, S. The Fab fragments of monoclonal IgG to a merozoite surface antigen inhibit *Plasmodium knowlesi* invasion of erythrocytes. *Mol. Biochem. Parasitol.* 13, 187-199 (1984).
15. Marshall, V.M., Zhang, L., Anders, R.F. & Coppel, R.L. Diversity of the vaccine candidate AMA1 of *Plasmodium falciparum*. *Mol. Biochem. Parasitol.* 77, 109-113 (1996).
16. Oliveira, D.A. et al. Genetic conservation of the *Plasmodium falciparum* apical membrane antigen-1 (AMA1). *Mol. Biochem. Parasitol.* 76, 333-336 (1996).
17. Hodder, A.N. et al. The disulfide bond structure of *Plasmodium* apical membrane antigen-1. *J. Biol. Chem.* 271, 29446-29452 (1996).
18. Thomas, A.W., Waters, A.P. & Carr, D. Analysis of variation in PF83, an erythrocytic merozoite vaccine candidate antigen of *Plasmodium falciparum*. *Mol. Biochem. Parasitol.* 42, 285-287 (1990).
19. Escalante, A.A. et al. Polymorphism in the gene encoding the apical membrane antigen-1 (AMA1) of *Plasmodium falciparum*. X. Asembo Bay Cohort Project. *Mol. Biochem. Parasitol.* 113, 279-287 (2001).
20. Kocken, C.H. et al. Molecular characterization of *Plasmodium reichenowi* apical membrane antigen-1 (AMA1), comparison with *P. falciparum* AMA1, and antibody-mediated inhibition of red cell invasion. *Mol. Biochem. Parasitol.* 109, 147-156 (2000).
21. Thomas, A.W. et al. High prevalence of natural antibodies against *Plasmodium falciparum* 83 kilodalton apical membrane antigen (PF83/AMA1) as detected by capture- enzyme-linked immunosorbent assay using full-length baculovirus recombinant PF83/AMA1. *Am. J. Trop. Med. Hyg.* 51, 730-740 (1994).

22. Udhayakumar, V. et al. Longitudinal study of natural immune responses to the *Plasmodium falciparum* apical membrane antigen (AMA1) in a holoendemic region of malaria in western Kenya: Asembo Bay Cohort Project VIII. *Am. J. Trop. Med. Hyg.* 65, 100-107 (2001).
23. Hodder, A.N., Crewther, P.E. & Anders, R.F. Specificity of the protective antibody response to apical membrane antigen 1. *Infect. Immun.* 69, 3286-3294 (2001).
24. Deans, J.A. et al. Vaccination trials in rhesus monkeys with a minor, invariant, *Plasmodium knowlesi* 66 kD merozoite antigen. *Parasite Immunol.* 10, 535-552 (1988).
25. Collins, W.E. et al. Protective immunity induced in squirrel monkeys with recombinant apical membrane antigen-1 of *Plasmodium fragile*. *Am. J. Trop. Med. Hyg.* 51, 711-719 (1994).
26. Xu, H. et al. CD4+ T cells acting independently of antibody contribute to protective immunity to *Plasmodium chabaudi* infection after apical membrane antigen 1 immunization. *J. Immunol.* 165, 389-396 (2000).
27. Anders, R.F. et al. Immunization with recombinant AMA1 protects mice against infection with *Plasmodium chabaudi*. *Vaccine* 16, 240-247 (1998).
28. Amante, F.H., Crewther, P.E., Anders, R.F. & Good, M.F. A cryptic T cell epitope on the apical membrane antigen 1 of *Plasmodium chabaudi adami* can prime for an anamnestic antibody response: implications for malaria vaccine design. *J. Immunol.* 159, 5535-5544 (1997).
29. Crewther, P.E., Matthew, M.L., Flegg, R.H. & Anders, R.F. Protective immune responses to apical membrane antigen 1 of *Plasmodium chabaudi* involve recognition of strain-specific epitopes. *Infect. Immun.* 64, 3310-3317 (1996).
30. Narum, D.L., Ogun, S.A., Thomas, A.W. & Holder, A.A. Immunization with parasite-derived apical membrane antigen 1 or passive immunization with a specific monoclonal antibody protects BALB/c mice against lethal *Plasmodium yoelii yoelii* YM blood-stage infection. *Infect. Immun.* 68, 2899-2906 (2000).
31. Kocken, C.H. et al. High-level expression of *Plasmodium vivax* apical membrane antigen 1 (AMA1) in *Pichia pastoris*: strong immunogenicity in *Macaca mulatta* immunized with *P. vivax* AMA1 and adjuvant SBAS2. *Infect. Immun.* 67, 43-49 (1999).
32. Cody, C.L., Baraff, L.J., Cherry, J.D., Marcy, S.M. & Manclark, C.R. Nature and rates of adverse reactions associated with DTP and DT immunizations in infants and children. *Pediatrics* 68, 650-660 (1981).
33. Merck and Co., Inc. Recombivax HB® Product Insert. Whitehouse Station, NJ (2003).
34. Saul, A., Lawrence G., Smillie A., et al. Human Phase 1 vaccine trials of 3 recombinant asexual stage malaria antigens with Montanide ISA 720 adjuvant. *Vaccine* 17:3145-3159 (1999).
35. Stoute, J.A., Slaoui, M., Heppner, D.G., et al. A preliminary evaluation of a recombinant circumsporozoite protein vaccine against *Plasmodium falciparum* malaria. RTS,S Malaria Vaccine Evaluation Group. *N Engl J Med.* 336:86-91 (1997).

36. Stowers, A.W., Kennedy, M.C., Keegan, B.P., et al. Vaccination of monkeys with recombinant *Plasmodium falciparum* apical membrane antigen 1 confers protection against blood-stage malaria. *Infect Immun.* 70:6961-6967 (2002).
37. Jilg, W., Schmidt, M., & Deinhardt, F. Vaccination against Hepatitis B: Comparison of Three Different Vaccination Schedules. J Infect Dis. 160, 766-769 (1989).
38. Hadler, S.C., Alcala de Monzon, M., Lugo, D.R., & Perez, M. Effect of timing of hepatitis B vaccine doses on response to vaccine in Yucpa Indians. *Vaccine* 7, 106-110 (1989).
39. Hadler, S.C. & Margolis, H.S. Hepatitis B immunization: vaccine types, efficacy, and indications for immunization. *Curr Curr Clin Top Infect Dis*. 12, 282-308 (1992).
40. Dolo, A., Thera, M.A., Baby, M., et al. Strain-specific antibody responses to potential malaria vaccine antigens in Mali. *Am. J. Trop. Med. Hyg.* 69(3) Suppl:495 (2003).
41. Sidibe,S., Sacko,B.Y. & Traore,I. [Prevalence of serologic markers of the hepatitis B virus in pregnant women of Bamako, Mali]. *Bull. Soc. Pathol. Exot.* 94, 339-341 (2001).

Appendix A – Summary of Preclinical Studies for AMA1-C1

Study #	Purpose	Species	Dose & Regimen	Formulations Tested	Summary
SV-AMA1-AS1	Immunogenicity of AMA1 FVO vs. AMA1 3D7	New Zealand White Rabbits Male (n=10)	50 µg in 0.5 mL on Days 0, 21 & 42 IM	Montanide ISA 720	AMA1 FVO + 3D7 produced significant immune response to both proteins. Each protein induced greater protection against homologous than heterologous protein. No adverse events reported.
AP-5005-00 SVP01-0064	Compare immunogenicity of AMA1 FVO & 3D7 separately and in combination	New Zealand White Rabbits Male (n=20)	25 µg total protein in 0.5 mL on Days 0 & 28 IM	Montanide ISA 720	AMA1 FVO + 3D7 produced significant immune response to both proteins. Separately, the proteins produced greater immune response to homologous than heterologous protein. No local or systemic adverse events reported.
AP-5006-00 SVP01-0081 SVP01-0106	Immunogenicity of AMA1 FVO/3D7 mixture in different adjuvants	New Zealand White Rabbits Male & Female (n=25)	20 µg total protein in 0.5 mL on Days 0 & 28 IM	Alhydrogel® Montanide ISA 720 MF59 QS21 Alhydrogel® + CpG 10105	AMA1 immunogenic in all adjuvants tested. No local or systemic adverse events reported.
1182-104	current Good Laboratory Practice toxicity & immunogenicity of AMA1-C1	New Zealand White Rabbits, Male & Female (n=24)	80 µg or placebo in 0.5 mL IM Days 1, 29, 50, & 71	Alhydrogel®	Antigen-specific immune responses to the antigen detectable which increased over time. Comparable responses to both components of the mixture. No adverse events observed.
AP-5009-00	Compare immunogenicity of AMA1-C1 dose and adjuvants	BALB/c mice Female (n=120)	1, 3, 10 µg total protein in 0.1 mL Days 0 & 28 IM	Alhydrogel®, Montanide ISA 720 QS21 Alhydrogel® + RC529 Alhydrogel® + CpG 10105	No adverse events in mice immunized with AMA1-C1/Alhydrogel®. AMA1-C1 immunogenic at all doses, in all adjuvants.
AP-5010-01	Immunogenicity/ baseline potency of AMA1-C1 (20 µg/0.5 mL formulation)	BALB/c mice Female (n=48)	0.3, 1, 3, 10 µg in 0.25 mL on Days 0 & 28 IP	Alhydrogel®	AMA1-C1 immunogenic even at low doses. AMA1-C1 baseline potency established. No local or systemic adverse events reported.
AP-5013-00	Immunogenicity of low dose AMA1-C1	BALB/c mice Female (n=90)	0.001, 0.01, 0.1, & 1 µg in 0.05 mL on Days 0 & 28 IM	Alhydrogel® CpG 1826 Saline alone Montanide ISA 720	No adverse events reported for mice receiving AMA1-C1/Alhydrogel®. Mouse that received 0.001 µg AMA1-C1 + Montanide ISA 720 found dead immediately following second immunization.
AP-5015-00	Immunogenicity and baseline potency of low dose AMA1-C1 (20 µg/0.5 mL formulation)	BALB/c mice Female (n=50)	0.01, 0.03, 0.1, 0.3, & 1 µg in 0.25 mL on Days 0 & 28 IP	Alhydrogel®	3 mice found dead 8 days after first immunization (2 naïve mice, and 1 in the 0.03 µg AMA1-C1 group), sent for necropsy. Cause of death may be related to inanition, and dehydration – food type changed. No other local or systemic adverse events reported. Dose response observed by ELISA.

Study #	Purpose	Species	Dose & Regimen	Formulations Tested	Summary
AP-5022-00	Stability of AMA1-C1 at 6 months (5, 20 & 80 µg/0.5 mL formulation)	BALB/c mice Female (n=180)	0.01, 0.03, & 0.1 µg in 0.25 mL on Days 0 & 28 IP	Alhydrogel®	MVDB-formulated product stable and potent at 6 months.. No local or systemic adverse events reported.
AP-5045-00	Stability of AMA1-C1 at 6 months (5, 20 & 80 µg/0.5 mL formulation)	BALB/c mice Female (n=240)	0.01, 0.03, & 0.1 µg in 0.25 mL on Days 0 & 28 IP	Alhydrogel®	NIH Pharmacy-formulated product stable and potent at 6 months. 1 mouse died minutes after injection; necropsy concluded death due to hemopericardium from injection.
AP-5029-00	Immunogenicity/baseline potency of AMA1-C1 (5, 20, & 80 µg/0.5 mL formulation)	BALB/c mice Female (n=180)	0.01, 0.03, 0.1, 0.3, & 1 µg in 0.25 mL on Days 0 & 28 IP	Alhydrogel®	NIH Pharmacy-formulated drug product equivalent in potency at baseline to MVDB reference antigen. No local or systemic adverse events reported.
AP-5044-00	Immunogenicity of AMA1 FVO, AMA1 3D7, & AMA1-C1	BALB/c mice Female (n=60)	0.03 & 1 µg total protein in 0.2 mL on Days 0 & 28 IP	Alhydrogel®	No adverse events reported. AMA1 3D7+FVO more immunogenic than either alone.
AP-5031-00	Immunogenicity of AMA1-C1	Rats (*Rattus norvegicus*) Female (n=24)	0.9 & 9.0 µg total protein in 0.1 mL on Days 0 & 28 IM	Alhydrogel® Alhydrogel® + CpG 10105	No adverse events reported. AMA1-C1+CpG more immunogenic than AMA1-C1/Alhydrogel®
AP-5021-00	Immunogenicity of AMA1-C1	Guinea pigs Female (n=45)	0.1 & 10 µg total protein in 0.2 mL on Days 0 & 28 IM	Alhydrogel® MF59	1 guinea pig died 3 days after 2nd immunization (1 µg group); necropsy showed hemopericardium due to phlebotomy of anterior vena cava. MF59 more immunogenic than Alhydrogel®.
AV010611	Safety, immunogenicity & efficacy of AMA1 FVO	*A. vociferans*, Male & Female (n=32)	100 µg subcutaneous in 0.5 mL on Days 0, 21, & 42	CFA & Montanide ISA 51	AMA1 FVO immunogenic and significant protection against homologous parasite challenge. No deviations in weight among the groups. No excess injection-site reactions beyond what was expected.
AP-5008-00	Safety & immunogenicity of AMA1 FVO/3D7 combination vs. AMA1 FVO and AMA1 3D7 alone	Rhesus monkeys *(Macacca mulatta)*, Male & Female (n=30)	25 µg in 0.5 mL on Days 0, 28, & 180 IM	Alhydrogel®, Montanide ISA 720 QS21, & Alhydrogel® + CpG 10105	AMA1 3D7 & FVO immunogenic; significant cross-reactivity by ELISA between 3D7 & FVO. No clinically significant local or systemic adverse events reported.
AP-5041-00	Supplement to AP-5008-00	Rhesus monkeys (Macacca mulatto), Male & Female (n=15)	25 µg in 0.5 mL on Day 360 IM	Montanide ISA 720	No local or systemic adverse events reported. Similar Ab titers after 1-year boost compared to 6-month boost.
AP-5020-00	Safety & immunogenicity of AMA1-C1 on Alhydrogel® and MF59 in rhesus	Rhesus monkeys *(M mulatta)*, Male (n=10)	10 µg in 0.5 mL on Days 0, 28, & 180 IM	Alhydrogel® MF59	No clinically significant local or systemic adverse events reported. No deviations in weight among the groups. AMA1-C1/Alhydrogel® more immunogenic than MF59.

IM = intramuscular
IP = intraperitoneal
SC = subcutaneous
CFA = Complete Freund's adjuvant
Ab = antibody

Appendix B – Map of Donéguébougou Village

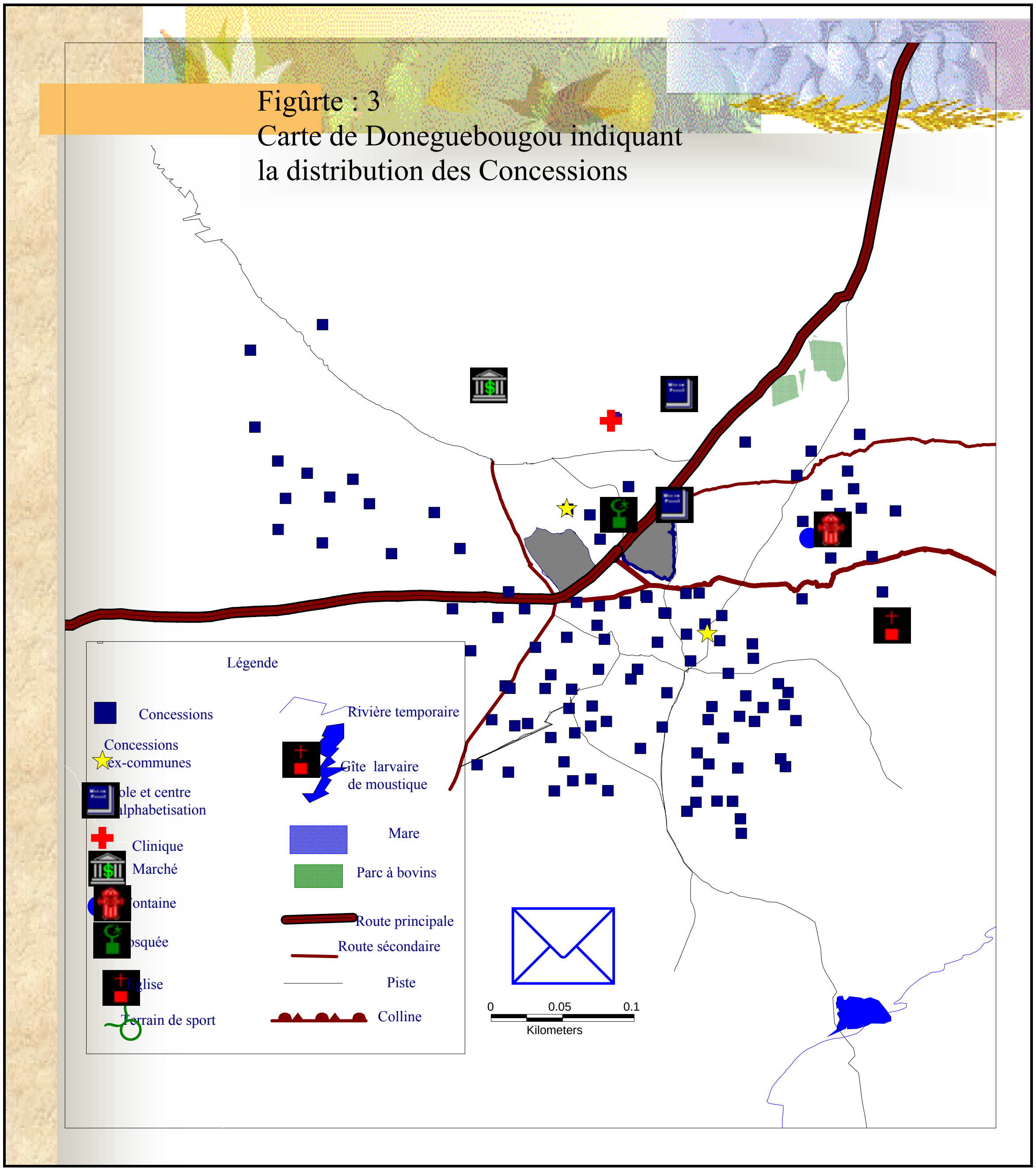

Appendix C – Malaria Vaccine Consent Comprehension Exam

MALARIA COMPREHENSION EXAM

Double-blind, randomized, controlled Phase 1 Study of the Safety and Immunogenicity of AMA1-C1/Alhydrogel® Vaccine for *Plasmodium falciparum* Malaria, in Semi-immune Adults in Donéguébougou, Mali

____________________ ______________________________

Census ID # Name (first, last)

1. As part of the study, you'll be injected with a live malaria parasite…...………….T F
2. There is a chance you could get sick from this vaccine……………..……………..T F
3. Women enrolled in this study should not become pregnant up until 1 month after the last shot….………………………………………………………….….….…T F
4. If you change your mind about being in the study after you are vaccinated, you can withdraw your consent……..………………………….…………….….…T F
5. This vaccine has been given to hundreds of people already, so we know it is completely safe……………………………………………..…….….………..….T F
6. You'll have your blood drawn as part of this study……………………………..….…T F
7. You'll get 3 vaccinations in this study……………………………………….….….…T F
8. If you feel sick during the study, you shouldn't tell anyone…………………....….…T F
9. If you join the study, you need to be followed in our clinic for 18 months….…..….T F
10. Everybody in this study will get the same kind of vaccine……………………….….…T F

- Total number correct before review……………….……..……… ___
- Total number correct after review…………………………….. ___

Reviewed by ______________________________ Date ___/___/___

Volunteer signature _________________________ Date ___/___/___

Witness signature __________________________ Date ___/___/___

Appendix D – Schedule of Visits

Procedures	Blood Volume	Day	Pre	0	1	2	3	7	14	28	29	30	31	35	42	60	90	120	150	180	270
Complete History/Physical			X																		
Obtain Informed Consent			X																		
Interim Clinical Evaluation				X	X	X	X	X	X	X	X	X	X	X	X	X	X	X	X	X	X
CBC	5 mL		X	X			X	X	X	X			X	X	X	X	X	X	X	X	X
ALT	5 mL		X	X			X		X	X			X		X						X
Creatinine			X	X			X		X	X			X		X						X
Urinalysis			X																		
Urine pregnancy test (females)			X	X						X											
HCV ELISA	5 mL		X																		
HBsAg ELISA			X																		
**VACCINATION**				**X**						**X**											
Anti-AMA1 antibody ELISA	5 mL			X					X	X					X		X		X	X	X
Growth inhibition assay	15 mL			X[1]											X					X	
B cell analysis	10 mL			X			X	X	X	X			X	X	X					X	
Filter paper blood collection				X					X	X					X	X	X	X	X	X	X
**Blood Volume (mL)**			15	55			20	15	25	25			20	15	40	5	10	5	10	35	15
**Total Blood Volume (mL)**			15	70			90	105	130	155			175	190	230	235	245	250	260	295	310

[1] Blood volume will be doubled for GIA on Study Day 0.

Appendix D – Schedule of Visits (continued)

**Procedures**	**Blood Volume**	**Day**	360	361	362	363	367	374	390	420	450	480	510	540
Complete History/Physical														
Obtain Informed Consent														
Interim Clinical Evaluation			X	X	X	X	X	X	X	X	X	X	X	X
CBC	5 mL		X			X	X	X	X	X	X	X	X	X
ALT	5 mL		X			X		X						
Creatinine			X			X		X						
Urinalysis														
Urine pregnancy test (females)			X											
HCV ELISA	5 mL													
HBsAg ELISA														
**VACCINATION**			**X**											
Anti-AMA1 antibody ELISA	5 mL		X					X		X		X		X
Growth inhibition assay	15 mL		X					X						X
B cell analysis	10 mL		X			X	X	X						X
Filter paper blood collection			X					X	X	X	X	X	X	X
**Blood Volume (mL)**			40			20	15	40	5	10	5	10	5	35
**Total Blood Volume (mL)**			350			370	385	425	430	440	445	455	460	495

Appendix E – Toxicity Table for Grading Laboratory Adverse Events

These tables are to be used to assess laboratory adverse events for those tests to be performed as part of the AMA1-C1 malaria vaccine clinical trial protocol.

ABBREVIATIONS: Abbreviations utilized in the Table:
ULN = Upper Limit of Normal LLN = Lower Limit of Normal

ESTIMATING SEVERITY GRADE

GRADE 1 **Mild:** no effect on activities of daily living; no medical intervention/therapy required

GRADE 2 **Moderate:** partial limitation in activities of daily living (can complete $\geq$ 50% of baseline); no or minimal medical intervention/therapy required

GRADE 3 **Severe:** activities of daily living limited to < 50% of baseline; medical evaluation/therapy required

GRADE 4 **Life-threatening (Serious Adverse Event):** life threatening, hospitalization required, persistent or significant disability/incapacity, resulting in death, or other important medical event that may require medical or surgical intervention to prevent one of the outcomes listed above

**HEMATOLOGY**				
	Grade 1	Grade 2	Grade 3	Grade 4
Hemoglobin<br>Males<br>Females	 9.5 – 10.5 g/dL<br>8.5 – 9.5 g/dL	 8.0 – 9.4 g/dL<br>7.0 – 8.4 g/dL	 6.5 – 7.9 g/dL<br>5.5 – 6.9 g/dL	 < 6.5 g/dL<br>< 5.5 g/dL
Platelets	75,000 - 99,999/mm^3	50,000 - 74,999/mm^3	20,000 - 49,999/mm^3	<20,000/mm^3
WBCs	11,500 – 13,000/ mm^3	13,000 - 15,000 /mm^3	15,000 - 30,000/mm^3	>30,000 or <1,000 /mm^3
Absolute Granulocyte Count	1000 -1500/mm^3	750-999/mm^3	500-749/mm^3	<500/mm^3

**CHEMISTRIES**				
	Grade 1	Grade 2	Grade 3	Grade 4
Creatinine	1.1 - 1.5 x ULN	1.6 - 3.0 x ULN	3.1 - 6 x ULN	> 6 x ULN or dialysis required
ALT	1.25 - 2.5 x ULN	2.6 - 5 x ULN	5.1 - 10 x ULN	> 10 x ULN

**URINALYSIS**				
	Grade 1	Grade 2	Grade 3	Grade 4
Proteinuria	2+<br>or<br>500 mg - 1 gm loss/day	3+<br>or<br>1- 2 gm loss/day	4+<br>or<br>2-3.5 gm loss/day	nephrotic syndrome<br>or<br>> 3.5 gm loss/day
Hematuria	5-10 rbc/hpf or 2+	>10 rbc/hpf or 3+	gross, with or without clots, OR red blood cell casts	requires hospitalization
